# 2D Prediction of the Nutritional Composition of Dishes from Food Images: Deep Learning Algorithm Selection and Data Curation Beyond the Nutrition5k Project

**DOI:** 10.3390/nu17132196

**Published:** 2025-06-30

**Authors:** Rachele Bianco, Sergio Coluccia, Michela Marinoni, Alex Falcon, Federica Fiori, Giuseppe Serra, Monica Ferraroni, Valeria Edefonti, Maria Parpinel

**Affiliations:** 1 Department of Medicine—DMED, Università degli Studi di Udine, 33100 Udine, Italy; bianco.rachele@spes.uniud.it (R.B.); federica.fiori@uniud.it (F.F.); maria.parpinel@uniud.it (M.P.); 2 Branch of Medical Statistics, Biometry and Epidemiology “G. A. Maccacaro”, Department of Clinical Sciences and Community Health, Dipartimento di Eccellenza 2023-2027, Università degli Studi di Milano, 20133 Milano, Italy; sergio.coluccia@unimi.it (S.C.); michela.marinoni@unimi.it (M.M.); monica.ferraroni@unimi.it (M.F.); 3 Department of Mathematics, Computer Science and Physics—DMIF, Università degli Studi di Udine, 33100 Udine, Italy; falcon.alex@spes.uniud.it (A.F.); giuseppe.serra@uniud.it (G.S.); 4 Fondazione IRCCS Ca’ Granda Ospedale Maggiore Policlinico, 20122 Milano, Italy

**Keywords:** deep learning, dietary assessment tools, energy prediction, food image recognition, frame filtering, macronutrients prediction, mass prediction, Nutrition5k, portion size correction, 2D prediction of nutritional composition

## Abstract

**Background/Objectives**: Deep learning (DL) has shown strong potential in analyzing food images, but few studies have directly predicted mass, energy, and macronutrient content from images. In addition to the importance of high-quality data, differences in country-specific food composition databases (FCDBs) can hinder model generalization. **Methods**: We assessed the performance of several standard DL models using four ground truth datasets derived from Nutrition5k—the largest image–nutrition dataset with ~5000 complex US cafeteria dishes. In light of developing an Italian dietary assessment tool, these datasets varied by FCDB alignment (Italian vs. US) and data curation (ingredient–mass correction and frame filtering on the test set). We evaluated combinations of four feature extractors [ResNet-50 (R50), ResNet-101 (R101), InceptionV3 (IncV3), and Vision Transformer-B-16 (ViT-B-16)] with two regression networks (2+1 and 2+2), using IncV3_2+2 as the benchmark. Descriptive statistics (percentages of agreement, unweighted Cohen’s kappa, and Bland–Altman plots) and standard regression metrics were used to compare predicted and ground truth nutritional composition. Dishes mispredicted by ≥7 algorithms were analyzed separately. **Results**: R50, R101, and ViT-B-16 consistently outperformed the benchmark across all datasets. Specifically, when replacing it with these top algorithms, reductions in median Mean Absolute Percentage Errors were 6.2% for mass, 6.4% for energy, 12.3% for fat, and 33.1% and 40.2% for protein and carbohydrates. Ingredient–mass correction substantially improved prediction metrics (6–42% when considering the top algorithms), while frame filtering had a more limited effect (<3%). Performance was consistently poor across most models for complex salads, chicken-based or eggs-based dishes, and Western-inspired breakfasts. **Conclusions**: The R101 and ViT-B-16 architectures will be prioritized in future analyses, where ingredient–mass correction and automated frame filtering methods will be considered.

## 1. Introduction

Traditional dietary assessment methods such as 24 h recalls, food frequency questionnaires, and dietary records remain widely used, but they face persistent challenges including recall bias, underreporting [1,2,3], and cultural variability that affect accuracy of reporting, especially when considering diverse dietary contexts [4]. In recent years, artificial intelligence (AI) has emerged as a powerful tool to overcome these limitations by enabling real-time, automated, and more precise dietary assessments [5,6]. This shift holds significant implications for public health, policy-making, and personalized nutrition strategies. Accurate dietary data is crucial to assessing the relationship between diet and disease, as well as to proposing tailored dietary recommendations to reduce the global burden of malnutrition and chronic diseases [7,8,9]. In personalized nutrition, accurate dietary assessments allow customization of dietary recommendations, which are tailored to individuals’ specific health needs, genetic predispositions, and lifestyle factors [5,10]. This is especially crucial for managing chronic conditions.

Despite the advancements made, food image recognition remains a complex task in computer vision due to various factors: image quality and setting [e.g., distance from which the image was taken and occlusions (i.e., presence of a food partially/totally hiding another one)], variability in food appearance (e.g., loss of visual information during food preparation, similarity between different foods, and different appearance of the same food across multiple images), and algorithmic limitations (e.g., multiple kernel learning, pairwise local features, and the bag-of-features model) [11,12].

Unlike traditional algorithms that rely on manually crafted features, deep learning (DL), a subset of AI, has demonstrated superior performance by automatically learning hierarchical features from raw data. This is especially evident with the use of Deep Convolutional Neural Networks and Vision Transformers (ViTs) [13], as well as with the integration of language supervision for multimodal image classification [14]. When applied to food images [12,15,16,17], DL outperforms traditional algorithms in extracting features from both generic [18] and food-specific images [12], eventually integrating vision–language models into this process [19]. In addition to estimating nutritional values [20], DL has been applied to generate realistic food images [21], create recipes [22], recognize flavors [23], and adapt recipes across cultures [24]. These advancements often build upon classical computer vision problems. Specifically, these models are capable of the following: (1) segmenting visual regions targeting specific food components [25]; (2) identifying ingredients [26] and linking them to recipes [21,22]; (3) estimating portion sizes and related volumes [12,27]. By capturing these fine-grained details, DL models can achieve higher accuracy [28,29,30,31]—particularly when trained on large and diverse datasets [32,33,34,35,36], including the new benchmark MetaFood3D [37]—and provide scalable solutions for assessing food intake.

One key example is the US Nutrition5k project [38], which introduced the first large-scale, open-access dataset of nearly 5000 images and metadata of generic food dishes (i.e., dishes that are not necessarily from a pre-determined set) with known nutritional content. Using robotic automation and multimodal data [i.e., Red Green Blue (RGB) video, depth images, ingredient weights, and high-accuracy nutritional values from the US Food Composition Database (FCDB) [39]], the project utilized Deep Convolutional Neural Networks for 2D direct prediction of mass and nutritional composition of dishes. This dataset has become a cornerstone for advancing DL-based dietary assessment and opens possibilities for testing model generalization across diverse experimental settings of data quality and culinary traditions, including Italian food.

The success of AI models heavily depends on high-quality, well-curated data. Preprocessing—which involves handling missing values, outliers, and inconsistencies—is vital to ensuring reliable model performance [40,41]. Nutritional differences across countries, shaped by agriculture practices, processing methods, and culinary traditions, pose an additional challenge. Variations in FCDBs, methodology of data collection, their curation standards, and completeness [42,43,44] can limit model transferability and accuracy when applied in new contexts. To enhance robustness and cross-cultural applicability, AI models must be trained on diverse datasets with accurate annotations. Poor labeling can propagate errors and obscure model performance. Therefore, data integrity, cultural representativeness, and comprehensive documentation are critical to building AI systems that support reliable and scalable dietary assessment within and across populations, including the Italian one [41].

In this study, we aim to use the US-based Nutrition5k project to evaluate the performance of various state-of-the-art DL algorithms, comparing them to the original benchmark algorithm presented in [38] in predicting mass, energy, and the macronutrient content from food images. In line with a previous article of our group [4], we will explore how different ground truth configurations—generated by combining data curation methods, such as ingredient–mass correction and frame filtering, with two country-specific FCDBs (the US FCDB used in Nutrition5k and the Italian FCDB)—affect performance of the selected algorithms. Finally, we will investigate if there are specific dishes consistently incorrectly predicted by most algorithms, and what common features they share.

## 2. Materials and Methods

### 2.1. Analytical Framework and Ground Truth Datasets for Analysis

This article built upon previous work on adapting the nutritional composition of Italian foods to align with the US Nutrition5k dataset for food image recognition [4]. Within this project, we have previously downloaded metadata files *cafe1* and *cafe2* (*dish_metadata_cafe1.csv* and *dish_metadata_cafe2.csv* files), which include the mass (in grams) and nutritional composition—energy (kcal), protein, fat, and carbohydrates (grams)—for the 5006 dishes included in the Nutrition5k project [45]. These values were calculated by summing ingredient-specific data sourced from the US Department of Agriculture (USDA) Food Composition Database [38,39]. To match each dish from Nutrition5k with its Italian nutritional composition, we followed these steps: (1) **exact or indirect matching** between dish-specific ingredients in Nutrition5k and food items in the Banca Dati di Composizione degli Alimenti [46]; (2) **manual data curation and handling of traces/missing values**, which included (a) removing “plate only” (i.e., empty) dishes, (b) identifying missing-name ingredients using dish images and supplementary files, (c) correcting dish mass values for ingredients with extreme weights, (d) imputing trace values [42], and (e) handling of missing values for food items in the Italian FCDB [42,47]; (3) **calculation of Italian-FCDB-specific nutritional values for each dish** in Nutrition5k by summing over the single-dish ingredients [4].

In the current application, video frames from the Nutrition5k dataset—including those from “plate only” dishes—were used to train and evaluate several standard DL algorithms for food image recognition. The goal was to predict the mass and nutritional composition of dishes in the test set. Since these outputs are continuous variables, the task was framed as a regression problem [12]. Training and performance evaluation were conducted across four distinct datasets, created from Nutrition5k by combining ingredient-mass correction with Italian-FCDB-specific nutritional values. Specifically, we have the following:

(1) **US FCDB—no correction**: Based on US-FCDB-specific nutritional values, without ingredient–mass correction;

(2) **US FCDB—correction**: Same as above, but with ingredient–mass correction applied;

(3) **IT FCDB—no correction**: Based on Italian-FCDB-specific nutritional values, which implied identifying and naming missing-name ingredients, as well as imputing traces and missing values for food items in the Italian FCDB, but without ingredient–mass correction;

(4) **IT FCDB—correction**: Same as above, but with ingredient-mass correction applied.

In a previous study [4], computed dish mass, energy, and macronutrient content were compared between datasets (2) and (4) to evaluate the impact of substituting US-FCDB with IT-FCDB nutritional values. That analysis excluded “plate only” dishes and involved identifying missing-name ingredients for proper matching. In the current study, we will expand on this by comparing predicted mass/nutritional values and model performance across all four datasets, focusing specifically on the effect of ingredient–mass correction—both within the US-FCDB-based datasets and the IT-FCDB-based ones. Our second goal will be to assess how this correction step influences the accuracy of various DL algorithms in predicting dish mass and nutritional content from food images. Figure S1 shows examples of food images from Nutrition5k and corresponding information on dish ID, observed mass, and nutritional values from the four available datasets.

### 2.2. Deep Learning for Food Image Recognition

Deep learning automatically and simultaneously performs both feature extraction and problem solving of complex tasks across various data types in an integrated way. It predominantly relies on artificial neural networks with multiple layers, where each layer’s weights are represented by real numbers. These weights are recursively optimized through the backpropagation algorithm, which calculates gradients for each batch of data (i.e., on a random subsample of observations of fixed size) during every epoch (i.e., iteration) with respect to a pre-specified loss function. The number of batches per epoch depends on the size of the training dataset (number of batches = training size/batch size). This process, also known as training, is repeated for a fixed number of epochs. As data passes through deeper layers, the model learns to extract increasingly complex features [48]. In the following subsections, we describe our pipeline, going from algorithm selection to performance evaluation.

#### 2.2.1. Splitting Data into Training, Validation, and Test Sets

To ensure reproducibility with results from Thames et al. [38], we adopted the same train/test split of dishes as in the Nutrition5k project, using the provided *rgb_train_ids.txt* and *rgb_test_ids.txt* files [45]. Initially, the training set contained 4059 dishes, and the test set had 709 dishes, all from the *dish_metadata_cafe1.csv* file [45]. However, 129 training dishes and 33 test dishes were excluded due to missing video frames from all cameras, leaving a final dataset of 3930 training dishes and 676 test dishes. Among these, 8 training dishes and 3 test dishes had undergone ingredient–mass corrections as reported in our previous publication [4]. Additionally, one “plate only” dish (*dish_1556575700*) was included in the training set, and another in the test set (*dish_1557861216*), in contrast to our prior analysis where these were discarded [4]. Notably, all dishes in the same incremental scan were included in the same split in Nutrition5k [38].

To optimize weights learning and evaluate generalization performance during training, we further split the training set into 90% for training and 10% for validation. To reduce the impact of stochastic factors—such as random initialization of the multi-layer perceptron (MLP) used for the problem-solving part as well as the random ordering of training data and batches—we implemented a k-repeated shuffled validation strategy, varying the 90/10 train/validation split across each of the k runs.

#### 2.2.2. Selecting the Architecture and Pre-Training Weights for the Automatic Feature Extraction

We scanned the official PyTorch repository of pre-trained models (PyTorch Vision Models 2.1.2) [49] to identify suitable architectures for predicting mass and nutrients from food images. Selection criteria included the following: (1) previous use on the Nutrition5k project [38]; (2) popularity (expressed as the number of citations) in multidisciplinary projects [50]; (3) dataset size vs. model complexity (expressed as the number of parameters and computational costs) [49]; (4) accuracy [49]. We first searched on PyTorch the algorithm proposed in Thames et al. [38], InceptionV2 (IncV2). Its updated version, InceptionV3 (IncV3) [51], is now available on the PyTorch repository and was assumed as the benchmark algorithm in our analysis [49]. Second, we selected Residual Network (ResNet) [52] and ViT [53] among the most highly cited algorithms used in interdisciplinary projects. Third, excessively large algorithms are unlikely to generalize effectively when trained on a relatively small dataset like Nutrition5k, as they require a substantially larger volume of data to learn meaningful representations. Fourth, we prioritized compact architectures (i.e., with a limited number of weights to train) that maintain competitive accuracy while ensuring computational efficiency and portability, particularly for potential deployment on mobile systems. Specifically, we focused on algorithms with a parameter count ≤ 75 million (vs. 27 million parameters considered in Nutrition5k) and top-1 accuracy on ImageNet ≥ 78.8% (78.8% is the percentage reported to be achieved by IncV3 [51]). Finally, we performed some preliminary experiments on a larger pool that also included ResNet18, ResNet34, and ViT-B-32. Overall, these considerations and the preliminary experiments performed suggested adding to the benchmark Inception_V3_IMAGENET1K_V1 the following algorithms with their pre-trained weights: ResNet101_IMAGENET1K_V2, ResNet50_IMAGENET1K_V2, and ViT_B_16_IMAGENET1K_SWAG_E2E_V1. Indeed, unlike the Nutrition5k project, which used the private dataset JFT-300M (300 million annotated generic images) [54] to pre-train IncV2, we relied on pre-trained weights from the open-source ImageNet (1 million annotated generic images) for each selected architecture. Detailed information on these architectures is provided in the Supplementary Material (Supplemental Methods section).

#### 2.2.3. Extracting and Preprocessing Frames

Each dish in Nutrition5k was associated with multiple video frames (up to four, one from each camera). To deal with multiple frames, we followed the procedure outlined in Nutrition5k for the training and validation phases, subsampling one out of five frames from each dish-specific video. This approach introduced variability across batches and epochs while reducing computational cost and memory usage. For testing, all available frames were used to enhance prediction robustness and minimize the impact of artifacts in the image capture process. The final prediction was obtained by averaging the results across all frames.

Once selected for training, validation, or testing, frames underwent preprocessing tailored to the neural network architecture used. This included resizing the image (to 232 × 232 for ResNet50 and ResNet101, 342 × 342 for IncV3, or 384 × 384 for ViT-B), extracting a central crop (to 224 × 224 for ResNet50 and ResNet101, 299 × 299 for IncV3, or 384 × 384 for ViT-B), scaling per-channel [0,255] pixel values to [0,1] using MinMax, and normalizing them by using the channel-specific mean and standard deviation from the ImageNet dataset [55].

#### 2.2.4. Setting the Architecture for Problem Solving

The pre-processed images were encoded into a fixed-size vector representation derived from the preceding feature extraction layer and used as the input to the fully connected prediction layer for problem solving. In detail, a 2048-dimensional vector was obtained after the global pooling layer for ResNet and IncV3, while a 768-dimensional vector was obtained from the “[class]” token for ViT [52,53]. To provide an efficient yet robust modeling of the relationship between food image and nutritional values, a MLP processed the previously extracted features to predict the dish mass, energy, and macronutrient content as described in the following.

On top of each feature extractor, we tested two variants of the MLP, here referred to as “2+1” and “2+2”, for the problem-solving part of the algorithm. In both variants, the input vector was first processed through two fully connected layers, interleaved by a nonlinear activation function (i.e., Rectified Linear Unit, ReLU). The resulting output was then processed in parallel through three trainable functions designed to predict mass, energy, and the three macronutrients together, as previously suggested in Thames et al. [38]. In the “2+1” variant, each of these functions was learned through a single fully connected layer for each of the three tasks, whereas in the “2+2” variant, each function underwent two fully connected layers interleaved with a ReLU activation. The “2+2” configuration was inspired by the MLP architecture used in Nutrition5k, whereas the “2+1” variant was tested to evaluate performance with fewer learned parameters for the predictors. Therefore, we compared eight algorithms (Table 1) with IncV3_2+2 which was taken as our benchmark algorithm.

#### 2.2.5. Setting the Optimization of the Loss Function for Feature Extraction and Problem Solving

Following the approach proposed in Nutrition5k [38], we selected the loss function given by the average of the single mean absolute errors (MAEs) calculated for the three tasks (i.e., prediction of mass, energy, and macronutrients, respectively), according to the following: $MAE=\frac{1}{N}\sum_{i=1}^{N}\left|{\hat{y}}_{i}-y_{i}\right|$, where ${\hat{y}}_{i}$ and $y_{i}$ represent the predicted and the ground truth values, respectively [38].

Training was conducted by assuming the following setting: 5 runs, 100 epochs, and an overall batch size of 64, but reduced to 32 for the ViT-B-16 algorithm, to mitigate memory limitations. The number of training epochs was fixed to ensure consistency and comparability across the various architectures and experimental datasets; specifically, preliminary results suggested that both training and validation loss typically reached a plateau well before epoch 100 (Figure S2, where two representative models were shown). Training relied on the Adam optimizer, with a learning rate of 0.001. Within each run, training was performed over the 100 epochs, and the weights achieving the best performance on the validation set across all epochs were selected for calculating the predictions on the test set. During training, the pre-trained weights for the ViT-B-16 architecture were kept frozen, meaning that only the MLP part of the network dedicated to problem-solving was trained. Indeed, evidence from the literature [53] suggested that ViTs typically require larger datasets to train effectively. Preliminary results additionally led to the worst performances when the full ViT backbone was trained.

#### 2.2.6. Setting the Performance Metrics for Problem Solving

Following the approach proposed in Nutrition5k [38], we evaluated the performance on the three regression tasks for each algorithm and run by using the (mean) absolute error as a direct value in its respective units (MAE) and as a percentage of the mean ground truth value (Mean Absolute Percentage Error, MAPE): MAPE = $\frac{1}{N}\sum_{i=1}^{N}\frac{\left|{\hat{y}}_{i}-y_{i}\right|}{\bar{y}}\times 100$, where $\bar{y}$ is the average of the *N* ground truth values. We also evaluated the Root Mean Squared Error (RMSE), which is a widely adopted metric in regression tasks: RMSE = $\sqrt{\frac{1}{N}\sum_{i=1}^{N}{\left({\hat{y}}_{i}-y_{i}\right)}^{2}}$.

We hypothesized that using a combination of performance metrics would provide a more comprehensive evaluation by capturing different error distributions and balancing the strengths and limitations of each metric [56]. In particular, we used the MAPE to facilitate performance comparisons across different tasks, as it is unitless and not tied to any specific measurement scale. On the other hand, RMSE may be useful for assessing the impact of ingredient–mass corrections, as its squared error formulation amplifies the influence of outliers and makes it particularly sensitive to extreme prediction errors. Finally, for each metric, target variable, and algorithm, performance was averaged over the five available runs.

#### 2.2.7. Predicting Mass, Energy, and Macronutrient Content with and Without Ingredient–Mass Correction and Italian Nutritional Value Calculation

After standardizing file formats, column names, and frame folder structures, we applied the processing steps outlined in previous sections to each ground truth dataset individually. These four ground truth datasets were defined by the presence or absence of ingredient–mass correction within both the US FCDB and IT FCDB frameworks: US FCDB—no correction, US FCDB—correction, IT FCDB—no correction, IT FCDB—correction. For each dataset, we generated dish-specific predictions for five target variables—mass, energy, protein, fat, and carbohydrates—using eight DL algorithms: IncV3_2+1, IncV3_2+2, R101_2+1, R101_2+2, R50_2+1, R50_2+2, ViT-B-16_2+1, and ViT-B-16_2+2. As for performance metrics, predictions were averaged over the five available runs. This resulted in a total of 160 predictions per dish (5 target variables × 4 datasets × 8 algorithms).

Subsequent statistical analyses were conducted on both ground truth (indicated as observed from now onwards) and predicted values for all dishes in the test set (*n* = 676).

#### 2.2.8. Sensitivity Analysis: Comparison of Predicted Versus Calculated Energy Content in 4-Task and 5-Task Scenarios

Nutritionists typically calculate total energy intake (in kcal) using the formula: 4 × protein (g) + 9 × fats (g) + 4 × carbohydrates (g) + 2 × fiber (g) + 7 × alcohol (g) [42,46]; in the case of carbohydrates expressed by monosaccharides equivalent, the conversion factor 4 is substituted by 3.75 [42,47]. Similarly, dish-specific energy content can be derived deterministically by summing macronutrient values multiplied by their respective conversion factors. This raises the question of whether energy content should be directly predicted by DL algorithms or instead calculated from predicted available macronutrients (without fiber and alcohol content because this information was not available in the Nutrition5k dataset). Moreover, since these tasks are interrelated, including energy prediction as a task may enhance the performance of DL models on macronutrient prediction—a common benefit observed in multi-task learning frameworks such as ours [57]. To explore this, we assessed two scenarios of calculated energy content obtained from the following: (1) 5-task algorithms, where prediction of energy was still performed but ignored, or (2) 4-task algorithms, where only mass and macronutrient content were predicted. Finally, we assessed the agreement between the three available scenarios: (1) energy predicted by the 5-task algorithms (i.e., *5-task predicted energy content*, main analysis), (2) energy calculated from macronutrients predicted by the 5-task algorithms (i.e., *5-task computed energy content*), and (3) energy calculated from macronutrients predicted by the 4-task algorithms (i.e., *4-task computed energy content*) across all datasets and algorithms. Performance metrics for scenarios (2) and (3) were computed and compared to those of the main analysis providing the 5-task direct prediction of energy content.

### 2.3. Statistical Analysis and Visual Inspection of Frames

We calculated summary statistics (minimum, 1st quartile, median, 3rd quartile, mean, standard deviation, and maximum values) for the dataset-specific distributions of the observed and predicted values of the target variables across all dishes in the test set. These statistics were also computed separately by algorithm for the predicted values. Finally, we also calculated the previous summary statistics on the merged dataset obtained by combining observed values for all the dishes from the four available datasets.

Dish-specific (raw, absolute) differences between predicted and observed values of the target variables were evaluated across datasets and algorithms by considering the following: (1) percentages of perfect, adjacent, and opposite agreement among quartile-based categories, along with unweighted Cohen’s kappa statistics, to take into account the possibility of agreement occurring by chance (with rules in a recent article [58] as follows: 0.01–0.39 as none to slight, 0.40–0.59 as weak, 0.60–0.79 as moderate, 0.80–1.00 as strong to very strong agreement) and (2) Bland–Altman plots and corresponding 95% limits of agreement.

To identify top-performing algorithms for comparison with our benchmark (IncV3_2+2), we calculated the median metrics for each target variable across the available datasets. Although all metrics were examined, the algorithm with the best median MAPE was first selected, or MAE in the event of a tie.

Furthermore, we defined incorrectly predicted dishes across target variables and datasets as those that satisfied both the following conditions for 7 or 8 DL algorithms: (1) exceeded the limits of agreement in the Bland–Altman plots and (2) had the highest 5% of absolute differences (regardless of sign) between observed and predicted values. These dishes were analyzed across target variables and datasets. A few of them were selected, together with one properly predicted benchmark dish, to illustrate the working dataset and the corresponding performance across the eight models. A nutritionist (R.B.) also conducted a visual inspection of dish frames from the four available cameras to identify potential issues with images and related ingredient labels that could distort performance and mask the true nutritional characteristics of the ingredients responsible for the poorest results.

Finally, in the sensitivity analysis, we evaluated the agreement between the following scenarios: (1) *5-task predicted energy content* (i.e., main analysis); (2) *5-task computed energy content*; and (3) *4-task computed energy content*, across datasets and algorithms, by calculating percentages of perfect, adjacent, and opposite agreement among quartile-based categories, and corresponding unweighted Cohen’s kappa statistics. Performance metrics were then compared across the three scenarios using median values across the available datasets.

## 3. Results

### 3.1. Descriptive Statistics on Observed and Predicted Values from the Test Set with and Without Ingredient–Mass Correction and Italian Nutritional Value Calculation

Table 2 presents summary statistics of observed values in the test set by target variable on the merged and single datasets. On the merged dataset, the median dish had a mass of 142 g, energy content of 164.5 kcal, 8.3 g of protein, 6.9 g of fat, and 11.3 g of carbohydrates (Table 2, right column). Across datasets and variables, the distributions were right-skewed, with median values consistently lower than the means. Ingredient–mass correction—specifically the adjustments involving asparagus (one dish) and olives (two dishes)—substantially reduced the maximum values in both US-FCDB and IT-FCDB datasets, except for protein content.

Table S1 reports summary statistics of predicted values from the test set by target variable, algorithm, and dataset. Frequency distributions of predicted values remained right-skewed. All algorithms tended to moderate extreme observed values for mass, energy, and macronutrients, pulling predictions toward the center of the distribution. As a result, maximum predicted values were similar across both corrected and uncorrected datasets, in contrast to the major differences seen in the observed values. Examples of predicted mass and nutritional composition for the different combinations of algorithms and available datasets were provided in Figure S1 for selected dishes.

### 3.2. Comparison Between Observed and Predicted Values from the Test Set with and Without Ingredient–Mass Correction and Italian Nutritional Value Calculation

Tables S2 and S3 provide percentages of perfect agreement and unweighted Cohen’s kappa coefficients obtained by cross-classifying dishes based on quartiles of observed and predicted values, respectively. Across available datasets and algorithms, perfect agreement was higher for mass (77–83%) and energy (76–82%) and lower for macronutrients: protein (62–78%), fat (65–78%), and carbohydrates (49–78%). When considering the same dataset, IncV3_2+2 (our benchmark algorithm) and IncV3_2+1 consistently showed the worst percentages across all target variables. For a given algorithm, perfect agreement was generally higher in the corrected datasets, with the exception of protein. Adjacent quartile switching occurred more frequently for macronutrients than for mass or energy, with ranges 17–23% for mass, 18–24% for energy, 19–34% for protein, 21–33% for fat, and 21–43% for carbohydrates. Opposite quartile misclassifications were rare, being <2.5%. Cohen’s kappa coefficients, which account for agreement by chance, ranged at 0.69–0.77 for mass, 0.67–0.76 for energy, 0.50–0.71 for protein, 0.53–0.71 for fat, and 0.32–0.71 for carbohydrates. These results indicate weak to moderate agreement, with higher values for mass and energy. As with the percentage agreement, kappa coefficients were typically higher for corrected datasets, again except for proteins, and lowest for the IncV3_2+2 and IncV3_2+1 algorithms.

Figures S3–S7 present Bland–Altman plots for each target variable, including 95% limits of agreement. These plots display the raw absolute difference between predicted and observed values on the test set against their mean. The mean difference (i.e., the bias) across datasets and algorithms ranged from −26.6 to −0.2 g for mass (Figure S3), from −50.7 to −2.1 kcal for energy (Figure S4), from −3.4 to −0.4 g for protein (Figure S5), from −4.2 to 0 g for fat (Figure S6), and from −3.5 to −0.6 g for carbohydrates (Figure S7). In all Figures S3–S7, the scatter around the bias line increased with higher values of the mean on the *x*-axis, indicating heteroscedasticity. When considering the same dataset, IncV3_2+2 and IncV3_2+1 generally provided wider limits of agreement across target variables (Figures S3–S7, first row vs. the remaining ones). After ingredient–mass correction, the mean difference was closer to zero for mass, energy, and fat content in both the US FCDB and IT FCDB datasets (Figures S3, S4 and S6, second vs. first column and fourth vs. third column from the left). Corresponding limits of agreement narrowed substantially by 6 to 10 times for mass, energy, and fat (Figures S3, S4 and S6, second vs. first column and fourth vs. third column from the left), and by 1 to 4 times for protein and carbohydrates (Figures S5 and S7; second vs. first column and fourth vs. third column from the left). After ingredient–mass correction, stronger reductions in the limits of agreement were also observed for the following architectures: R101 for mass and energy content (Figures S3 and S4, second row from the top vs. the remaining ones, second vs. first column, and fourth vs. third column from the left) and ViT-B-16 for protein and carbohydrates content (Figures S5 and S7, fourth row from the top vs. the remaining ones, second vs. first column, and fourth vs. third column from the left), in the absence of a clear indication for fat content (Figure S6, second vs. first column and fourth vs. third column from the left). IncV3_2+2 and IncV3_2+1 showed weaker reductions in the limits of agreement (Figures S3–S7, first row vs. the remaining ones, second vs. first column, and fourth vs. third column from the left).

### 3.3. Comparison Between Metrics with and Without Ingredient–Mass Correction and Italian Nutritional Value Calculation

**Figure 1:**
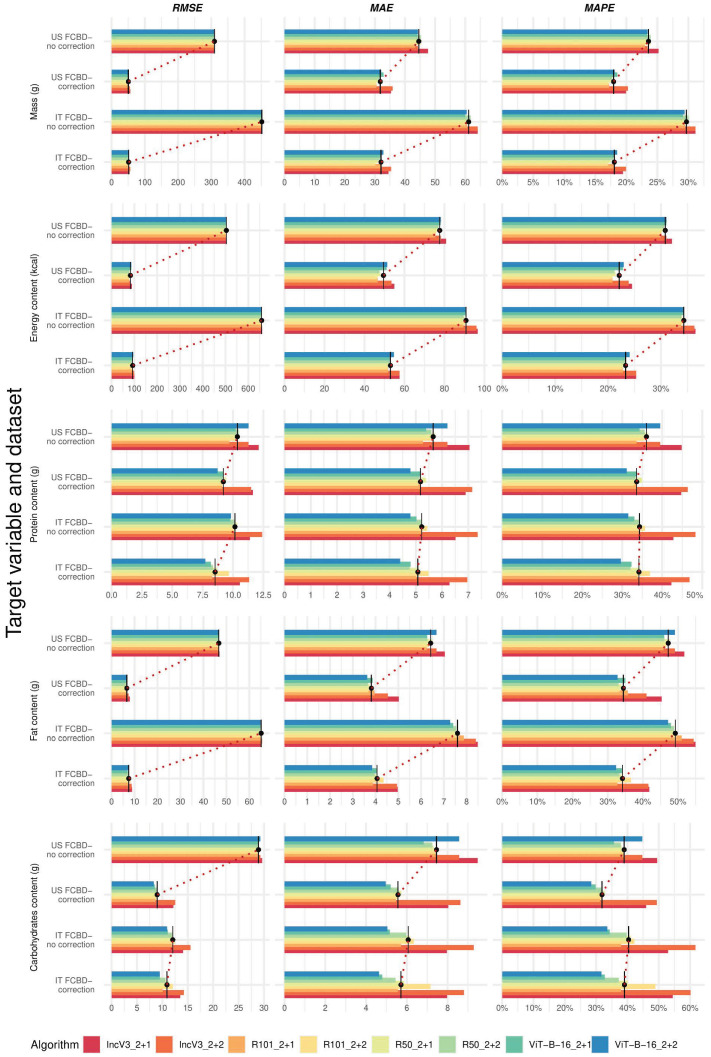
Performance metrics for single target variables, algorithms, and available datasets. Abbreviations: FCDB, Food Composition Database; MAE, Mean Absolute Error; MAPE, Mean Absolute Percentage Error; RMSE, Root Mean Squared Error (see Table 1 for algorithm name abbreviations and Figure 1 legend for the color scale). To improve readability, each subfigure displays performance metrics using its own scale, reflecting substantial variations in the metric’s range.

Figure 1 presents the median performance metrics for single target variables, algorithms, and datasets (details in Table S4). While providing a fair comparison across target variables, MAPE values ranged from 17.0% to 31.1% for mass, 20.7% to 36.5% for energy, 29.6% to 48.3% for proteins, 32.4% to 55.0% for fats, and 28.4% to 61.7% for carbohydrates across all datasets and algorithms (Table S4, upper panels). When considering the same dataset, IncV3_2+2 (our benchmark algorithm) and IncV3_2+1 generally exhibited the worst performance across target variables and metrics, although RMSE values were more similar than the other metrics (Figure 1, red colors (darker and lighter) vs. the remaining ones in each subfigure, and Table S4). Notably, when comparing corrected datasets to uncorrected ones for the same algorithm, the metrics were typically lower for the former, with the exception of protein; specifically, when comparing the top-performing algorithm on the corrected versus uncorrected dataset, accuracy gains in MAPE ranged from 5.6% (carbohydrates) to 41.6% (mass), both obtained within the IT FCDB. The detected effect was particularly strong for the RMSE, which is more sensible to the extreme values presented in the uncorrected datasets (Figure 1, dashed line in each subfigure, and Table S4).

### 3.4. Selecting the Best-Performing Deep Learning Algorithms

**Figure 2:**
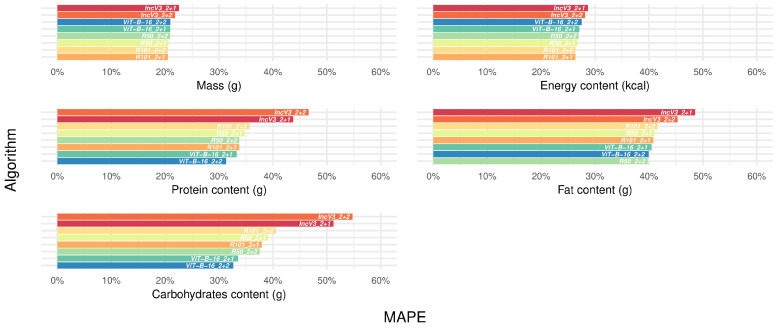
Median error, as measured by mean absolute percentage error, for single target variables and algorithms across datasets, before frame filtering. Abbreviations: MAPE, Mean Absolute Percentage Error (see Table 1 for algorithm name abbreviations and Figure 1 legend for the color scale).

Figure 2 presents the median MAPE for single target variables and algorithms, calculated over available datasets (details for additional metrics and available datasets in Table S4). The median error as measured by MAPE ranged from 20.5 to 22.6% for mass, from 26.4 to 28.7% for energy, from 31.3 to 46.6% for protein, from 39.7 to 48.6% for fat, and from 32.7 to 54.8% for carbohydrates (Table S4, lower panel). More generally, mass and energy content had more similar and lower error metrics compared to macronutrients, with carbohydrates roughly doubling the maximum error for mass and energy. Across all target variables and metrics, the IncV3_2+1 and IncV3_2+2 algorithms consistently performed worse than the others. Based on median MAPE and MAE, the top-performing algorithms were R101_2+1 for mass and energy, R50_2+2 for fats, and ViT-B-16_2+2 for protein and carbohydrates. When replacing the benchmark with these top algorithms, reductions in median MAPEs were observed: 6.2% for mass, 6.4% for energy, 12.3% for fat, and 33.1% and 40.2% for protein and carbohydrates, respectively. However, as the macronutrients were predicted in the same task, the final list of selected algorithms included R101_2+1 for mass and energy and ViT-B-16_2+2 for protein, fat, and carbohydrates content, together with the benchmark IncV3_2+2 (Table S4, lower panel).

### 3.5. Incorrectly Predicted Dishes Across Target Variables and Datasets: A Focus on Nutritional Characteristics of the Ingredients After Manual Frame Filtering

While looking for model-independent sources of error, 80 dishes were identified as incorrectly predicted by 7 or 8 DL algorithms (see Section 2.3). A manual frame-by-frame inspection of the images from the four cameras by a nutritionist (R.B.) revealed that 12 of these dishes had clear issues:

- 7 dishes (*dish_1563566909*, *dish_1566414291*, *dish_1566501575*, *dish_1566501594*, *dish_1566589933*, *dish_1563566939*, and *dish_1563566965*) showed mismatches between visible ingredients and the (lower number of) ingredients listed in the metadata, mostly leading to overestimated predictions; the only reasonable exception was represented by carbohydrates, where dish ingredients were not entirely captured by most available images;
- 5 dishes (*dish_1558630325*, *dish_1558720236*, *dish_1562703447*, *dish_1563389626*, and *dish_1566838351*) suffered from image quality problems in all frames, including poor framing, blurriness, cropping, operator hands/feet in the frame, and background elements (e.g., chairs, tables, cables, and phones).

These 12 problematic dishes were excluded from further analysis, resulting in a refined test set of 664 dishes, of which 68 remained consistently mispredicted (number of frames, median: 32, IQR: 29–37, range: 24–819) (Table S5). After filtering frames for all incorrectly predicted dishes, a median of 39% (IQR: 24–50%) of the original frames remained, resulting in a median of 12.5 frames per dish (IQR: 8.75–18.25) (Table S5).

**Figure 3:**
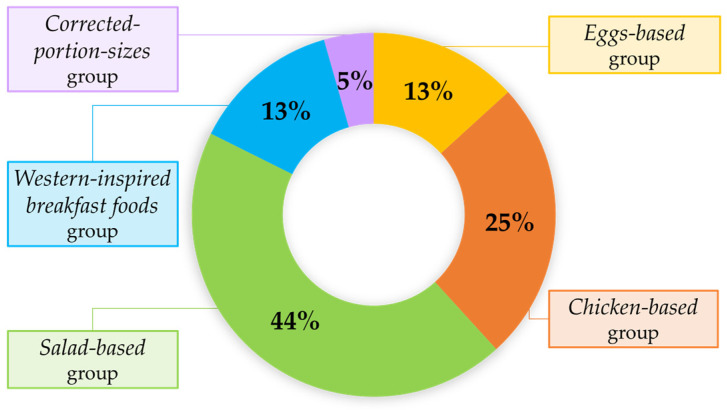
Frequency distribution of incorrectly predicted dishes by groups based on similarity in ingredient content and the presence of ingredient–mass correction.

Besides the so-called *Corrected-portion-size* group (5%), we grouped the remaining incorrectly predicted dishes based on ingredient similarity into the following categories (Figure 3 and Table S5):

- *Salad-based* group (44%): Complex dishes, with at least two vegetables (e.g., raw and/or cooked), two protein sources (e.g., fish and/or meat), two cereal types, herbs, and dressings (median number of ingredients per dish: 16, IQR: 11.25–20.5);
- *Chicken-based* group (25%): Dishes primarily composed of chicken (grilled, breaded, or sliced in a cold salad), simpler in composition, but sometimes visually ambiguous (median number of ingredients per dish: 4, IQR: 3–5);
- *Eggs-based* group (13%): Dishes featuring mainly scrambled eggs or boiled egg whites, with few ingredients, but often showing partial occlusion (median number of ingredients per dish: 5, IQR: 3–6);
- *Western-inspired breakfast foods* group (13%): Hybrid category featuring dishes with a limited number of ingredients, blending elements of both sweet (e.g., fruit, almonds, or brownies) and savory (e.g., potatoes, sausages, or cheese) breakfasts without a single dominant component (median number of ingredients per dish: 2, IQR: 1–3).

Finally, the *Corrected-portion-size* group (median number of ingredients per dish: 3, IQR: 2–3) included three dishes from the test set with known ingredient–mass corrections (as described in [4]). These dishes showed poor prediction performance in the uncorrected datasets only, but either across all target variables (*dish_1551567508*) or for protein and carbohydrates only (*dish_1551382179*); this depended on the corrected ingredient, as asparagus, among macronutrients, is a main source of protein and carbohydrates. Notably, reporting errors in Nutrition5k led to inconsistencies between listed ingredient masses and total dish mass for *dish_1551567573* and *dish_1551382179*, contributing to those dishes not being mispredicted in mass on the US FCDB—no correction dataset (Table S5).

### 3.6. Confirming the Best-Performing Deep Learning Algorithms After Manual Frame Filtering

**Figure 4:**
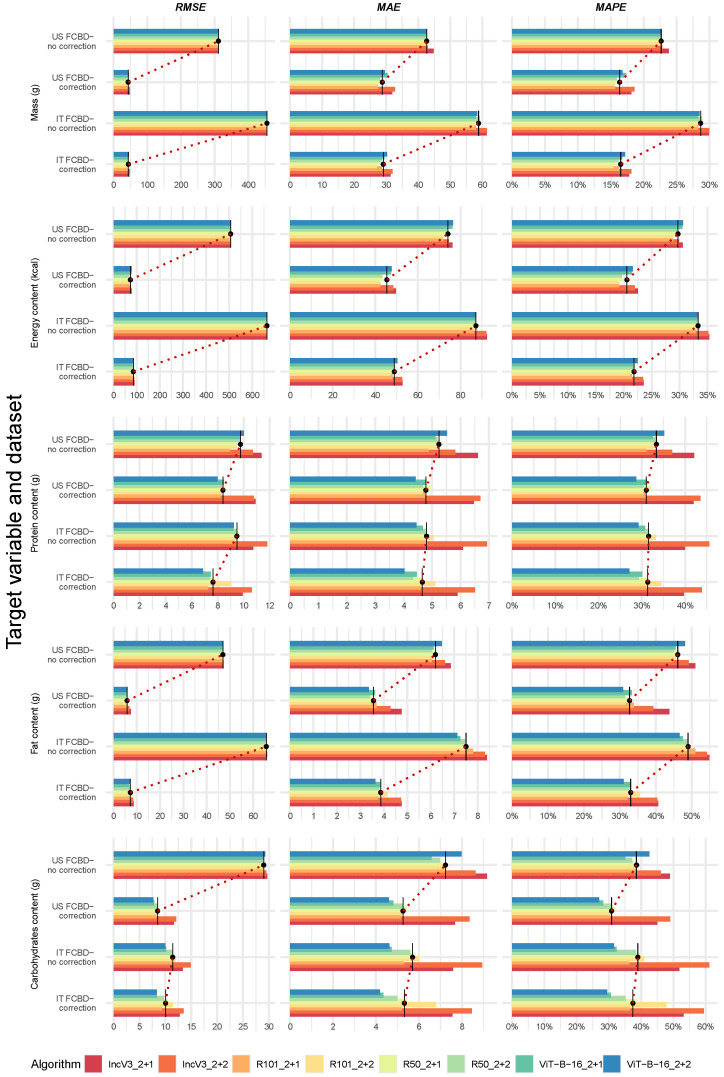
Performance metrics for single-target variables, algorithms, and available datasets, after frame filtering. Abbreviations: FCDB, Food Composition Database; MAE, Mean Absolute Error; MAPE, Mean Absolute Percentage Error; RMSE, Root Mean Squared Error (see Table 1 for algorithm name abbreviations and Figure 4 legend for the color scale). To improve readability, each subfigure displays performance metrics using its own scale, reflecting substantial variations in the metric’s range.

**Figure 5:**
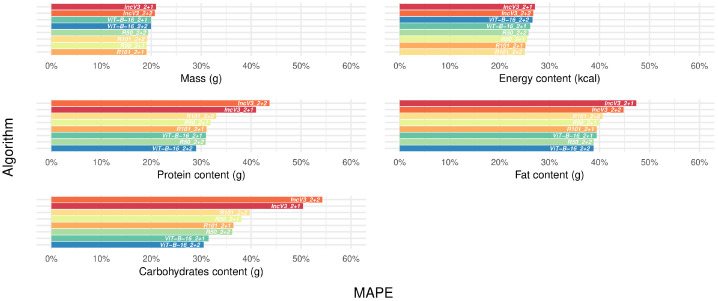
Median error, as measured by mean absolute percentage error, for single-target variables and algorithms across datasets after frame filtering. Abbreviations: MAPE, Mean Absolute Percentage Error (see Table 1 for algorithm name abbreviations and Figure 4 legend for the color scale).

Figure 4 and Figure 5 present the median performance metrics by target variable and algorithm, across individual datasets and overall, following manual frame filtering (details in Table S6). We observed the following effect of manual frame filtering: (1) Reduced Variation, Minimal Impact: frame filtering slightly narrowed the range of MAPE values across datasets and algorithms, but the improvements were modest—typically under 3% (Figure 4 and Table S6, upper panels); (2) Correction Still Matters: when considering the same algorithm, performance remained consistently better on corrected datasets compared to uncorrected ones for all metrics, but especially for the RMSE (Figure 4); (3) Consistent Underperformance of Benchmark: when considering the same dataset, IncV3_2+2 and IncV3_2+1 generally showed the worst metrics across target variables, except for RMSE (Figure 4, red colors (darker and lighter) vs. the remaining ones in each subfigure, and Figure 5); (4) Marginal Overall Gains: when datasets were merged, frame filtering led to slight reductions in median MAPE, with ranges as follows: 19.0–21.0% for mass, 25.0–27.1% for energy, 29.0–43.7% for protein, 38.8–47.4% for fat, and 30.5–54.2% for carbohydrates (Figure 5 and Table S6, lower panel); (5) Top Performers Confirmed: the top-one algorithms generally remained consistent with previous results, with R101_2+1 for mass, R101_2+2 for energy content, and ViT-B-16_2+2 for proteins, fats, and carbohydrates (Figure 5 and Table S6, lower panel). Overall, these results reinforced the superior performance of the R101 and ViT-B-16 architectures compared to the benchmark IncV3_2+2, even after accounting for image quality through manual frame filtering.

### 3.7. Sensitivity Analysis: Comparing Predicted Versus Calculated Energy Content Using the 4-Task and 5-Task Deep Learning Algorithms

When comparing predicted and calculated energy content using the 4-task and 5-task DL algorithms, the highest agreement was observed between the *5-task predicted* and *5-task computed* energy values. This pair showed the highest percentages of perfect agreement (59–94% on uncorrected datasets; 71–92% on corrected datasets) and lowest adjacent quartile switching, alongside moderate-to-strong agreement based on unweighted Cohen’s kappa across all datasets and algorithms. In contrast, the other two pairs (*5-task* vs. *4-task computed*, and *5-task predicted* vs. *4-task computed*) had lower but comparable agreement metrics, with perfect agreement generally between 60 and 80% on uncorrected/corrected datasets, and kappa values indicating weak-to-moderate agreement across datasets and algorithms. Across all datasets and investigated pairs of computed and predicted energy content, the IncV3_2+1 or IncV3_2+2 algorithms generally showed the worst agreement, whereas the ViT-B-16_2+1 and ViT-B-16_2+2 showed the best agreement according to both percentages of perfect agreement and Cohen’s kappa coefficients.

Restricting the sensitivity analysis to the 664 test dishes with potentially reduced frame counts confirmed previous findings. Specifically: (1) the *5-task predicted*–*5-task computed energy content* pair showed the highest agreement; (2) across all datasets and investigated pairs, IncV3_2+1 or IncV3_2+2 generally showed the worst, whereas ViT-B-16_2+1 or ViT-B-16_2+2 showed the best agreement (Tables S7 and S8). However, when the US FCDB—no correction dataset was considered, the agreement for the pair *5-task predicted*–*5-task computed energy content* substantially improved for the IncV3_2+2 and ViT-B-16_2+2 algorithms after frame filtering.

**Figure 6:**
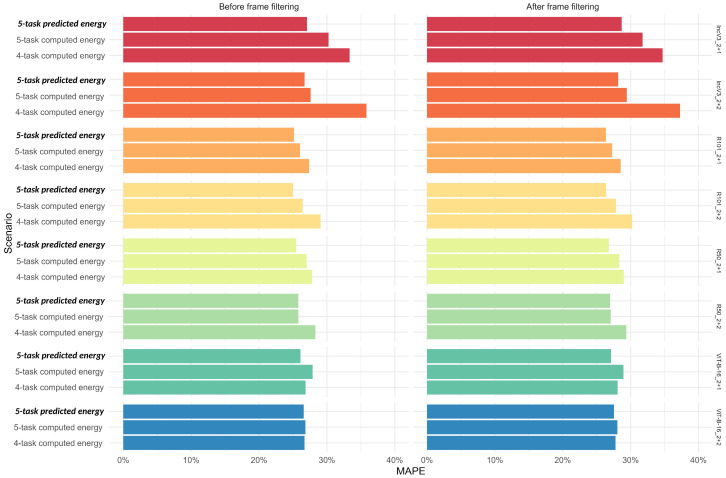
Sensitivity analysis on energy content: median error, as measured by mean absolute percentage error, of single algorithms (in rows) for the predicted and computed energy content scenarios under the 4-task and 5-task deep learning algorithms across datasets, before and after frame filtering (in columns). The scenario presented in the main analysis is indicated in bold and italics typeface. Abbreviations: MAPE, Mean Absolute Percentage Error (see Table 1 for algorithm name abbreviations and legends in Figure 1 and Figure 4 for the color scale).

Finally, compared to the *5-task predicted energy content* scenario from the main analysis, performance metrics for the other two scenarios were generally worse across all algorithms, with the exception of ViT-B-16_2+1 and ViT-B-16_2+2, which showed minimal differences (<7%). The *4-task computed energy content* scenario performed substantially worse than the *5-task computed energy content* one, with percentage differences from the main analysis reaching up to 34%, versus a maximum of 12% for the *5-task computed energy content* scenario. In contrast, mass and macronutrient predictions were more stable, showing <10% variation from the main analysis. The largest discrepancies occurred in fat prediction under the *4-task computed energy content* scenario, where differences reached 28% both before and after frame filtering (Figure 6 and Table S9).

## 4. Discussion

This article investigates the use of the Nutrition5k dataset—the largest and most diverse annotated dataset of complex, real-world dishes—for directly predicting the mass and nutritional composition of dishes using 2D images. The study addresses three key research questions: (1) Do standard and selected DL algorithms outperform the Nutrition5k benchmark when using the same train/test split, task setup, loss function, and performance metrics? (2) How does manual data curation, such as ingredient–mass correction and frame filtering, affect prediction accuracy and model performance? (3) Are there specific dishes consistently incorrectly predicted by most DL models, and what common features do these dishes share? To test generalizability, analyses were conducted using both the US FCDB from Nutrition5k and the Italian FCDB, aiding in the development of a DL-based dietary assessment tool for Italy. Our main results support the following conclusions: (1) All selected DL architectures outperformed the benchmark IncV3_2+2 or the related IncV3_2+1, both before and after ingredient–mass correction and frame filtering. (2) Adjusting extreme portion sizes for three test dishes improved prediction accuracy and model performance—except for protein content; frame filtering on the test set, however, had minimal impact. (3) Dishes frequently mispredicted across target variables and datasets tended to be complex salads, chicken-based meals, Western breakfast foods, and eggs-based dishes, in addition to those with corrected portion sizes. Finally, a sensitivity analysis comparing DL-predicted energy content versus energy contents calculated from macronutrients showed that direct energy prediction typically yielded better results.

All six selected algorithms outperformed the IncV3_2+2—an updated version of the IncV2 algorithm from the original Nutrition5k project—as well as the lighter IncV3_2+1. This superiority held true both before and after applying ingredient–mass correction and frame filtering. Preliminary comparisons of observed versus predicted values, conducted both before and after ingredient–mass correction and using various agreement measures, showed consistent results. This robustness across different conditions strengthens the case for updating the algorithm lineup in future studies. While the best-performing algorithm varied depending on the nutritional target variable, the R101 and ViT-B-16 architectures consistently showed strong performance and will be prioritized in future analyses. For a fair comparison with the IncV3_2+2 benchmark, configurations R101_2+2 and ViT-B-16_2+2 will be used. In addition, consistent with Thames et al. [38], we observed a decline in algorithm performance from predicting mass and energy to protein, carbohydrates, and fat. This trend likely reflects the increasing complexity of these tasks, with macronutrient prediction being more challenging than mass or energy estimation. Among macronutrients, fat predictions had the highest median errors, likely due to the difficulty in detecting fat-rich components like dressings from food images.

Manual data curation is known to enhance the performance metrics of DL algorithms [40,41]. In our case, correcting ingredient masses led to substantial gains in accuracy—reflected in improved perfect agreement percentages and Cohen’s kappa coefficients—and overall performance metrics. These improvements were consistent across most target variables, except for protein. This outcome is expected, as the corrected ingredients—olives and asparagus—primarily affected fat and carbohydrates predictions, in addition to mass [39,47]. We also evaluated manual frame filtering as a preliminary step toward future implementation of automated filtering, potentially extending to the training set. As expected, performance improvements were modest, which is reasonable given that filtering was limited to 80 mispredicted dishes out of 676 in the test set. However, our reproducible research pipeline intentionally treated manual frame filtering as a secondary analysis, applying it only to the test set—and specifically to the mispredicted subset—to maintain comparability with prior results from the Nutrition5k project [38]. Furthermore, this analysis allowed us to assess and document the considerable manual effort involved in frame filtering: a single researcher devoted 16 full working hours to meticulously review 80 dishes. Reporting this workload helps set a baseline for human involvement and informs the assessment of whether, and how, automated approaches could be usefully developed for future analyses.

In this study, we also investigated which dish characteristics might account for consistently poor performance across algorithms, target variables, and both corrected and uncorrected datasets. To understand sources of poor model performance, we defined specific criteria, analyzed individual dishes across datasets and target variables, and grouped them by ingredient similarity. Dishes frequently mispredicted—beyond those with corrected portion sizes—included complex salads, chicken-based meals, eggs-based dishes, and Western-style breakfasts. Salad-based dishes were especially challenging, often containing at least two types of vegetables, high-protein foods, cereals, herbs, and dressings, with a median of 16 ingredients per dish. The high ingredient count increased the likelihood of occlusion, and undetected dressings contributed to underestimations of fat content. Chicken- and eggs-based dishes, typically simpler in composition, were often underestimated in protein content, likely due to the visual similarity between chicken or eggs and the white bowls or plates they were served on, making recognition more difficult. This especially happened for boiled egg whites and sliced grilled chicken without skin, where it was materially impossible to distinguish food color from that of the background. Finally, Western breakfast dishes—though composed of fewer ingredients—still posed challenges. Some of these, such as dishes containing almonds or raw spinach, may benefit from incorporating volume estimates into predictions. For instance, inaccuracies in predicting almonds were likely linked to their placement on smaller plates, when alone, or alongside other masking ingredients. Overall, the consistent mispredictions observed across all model types suggest that certain types of dishes—particularly those with high visual complexity or ambiguous ingredient composition—remain systematically challenging for current nutrient prediction approaches. However, proper reporting of mass and nutritional information, a fair ingredient visibility, and more appropriate or standardized annotations could help models better predict the nutritional content of these consistently mispredicted dishes.

Finally, we examined the decision—also made in the Nutrition5k project [38]—to predict energy content as a separate task from mass and macronutrients, as part of a sensitivity analysis. Indeed, nutritionists typically calculate energy intake by summing macronutrient values multiplied by their respective conversion factors. This raises the question of whether DL algorithms should directly predict energy content or derive it from predicted macronutrient values. We observed a performance gradient, with algorithm metrics improving progressively from the *4-task computed energy content*, to the *5-task computed energy content*, and finally to the *5-task predicted energy content*. The poorer performance of the *4-task computed energy content*—mirroring a real-world, nutritionist-style calculation—can be attributed to error propagation due to the summation process of the macronutrients; given that algorithm performance is particularly weak for fat, inaccuracies in fat prediction likely contribute most significantly to the overall error in energy content calculation. The intermediate performance of the *5-task computed energy content*—representing a hypothetical scenario where energy prediction influences the macronutrient prediction but was ultimately excluded from the final output—may reflect a stabilizing effect of energy prediction. In this setup, energy prediction can partially compensate for macronutrient prediction errors, particularly those arising from image grounding issues. Consequently, the *5-task computed energy content* scenario is the most similar to the *5-task predicted energy content* from the main analysis, which should still be prioritized in future analyses.

This study has several strengths and limitations. A key strength lies in its multidisciplinary team—comprising informaticians, nutritionists, and medical statisticians—which enabled a comprehensive approach. In addition to standard aggregate-level performance metrics, this team applied dish-level statistical analyses, from standard procedures for outliers’ detection to measures of agreement and performance evaluation. These analyses revealed data quality issues in the Nutrition5k project, emphasizing the need for manual curation beyond standard nutritional database checks, which typically ensure that macronutrients, fiber, and ash sum to 100 [59]. Likewise, grouping mispredicted dishes helped uncover ingredient-related factors contributing to poor algorithm performance. Finally, the sensitivity analysis helped to assess if translating typical reasoning schemes of nutritionists into DL practice could improve performance and reduce computational effort. Crucially, curation, the analysis of mispredicted dishes, and the sensitivity analysis were made possible through close collaboration between expert nutritionists, medical statisticians, and informaticians. Another strength of this study is its strong emphasis on reproducibility, achieved by closely aligning each step with the Nutrition5k project, whenever possible. We adopted the same train/test split, frame selection criteria, task distribution, model architecture, loss function, and performance metrics. We also used an updated version of Nutrition5k’s benchmark algorithm for 2D direct prediction. When changes were introduced, we reported results from the original Nutrition5k analysis to ensure transparency and maintain comparability.

Among the limitations, the Nutrition5k dataset—though well designed—remains limited in scale and exhibits errors in image data, nutritional values, and ingredient labels. Our manual frame filtering experiment revealed that only a median of 39% of frames were retained per mispredicted dish, suggesting potential errors in the image data. Whether this rate is representative of the full dataset—or applicable to the training set—remains to be assessed through automated filtering methods. Furthermore, switching from the US to the Italian FCDB may have increased uncertainty in nutritional values due to the lack of standard USDA food IDs, complicating item matching. We also noted mismatches between some food images and their associated ingredient labels in Nutrition5k. While we verified nutritional values and ingredient labels using descriptive statistics, more advanced methods or alternative misprediction detection strategies could potentially uncover additional issues. Second, although fixed central cropping may have systematically excluded information located at the image periphery—thus resulting in the loss of important visual cues (e.g., side dishes, garnishes, or context elements that aid portion size estimation)—the preliminary resizing of the images has likely reduced the potential impact of this decision; in addition, random central cropping [18] on the training set might be more effective in image pre-processing. Third, the comparison of results across so many algorithms, datasets, and target variables might be difficult to manage, although results were very consistent across scenarios. Finally, while predicting nutritional composition directly from 2D food images is a valuable task—particularly in collaboration with expert nutritionists—there remains a scarcity of datasets that combine food images with full information on energy, macronutrients, and mass [26,33,35,36,60,61]. Exploring self-supervised representation learning methods, such as SimCLR [62] and SwAV [63], offers a promising direction. These approaches enable fine-tuning of models for nutritional prediction, without the need for extensive labeled data. Nevertheless, the limited availability of annotated datasets continues to hinder the generalizability of current findings.

## 5. Conclusions

In this work, we systematically benchmarked state-of-the-art DL architectures—including IncV3, R101, R50, and ViT-B-16—for nutrient prediction on the Nutrition5k dataset [38]. While all models outperformed those from the IncV3 family, we observed that performance gains were not uniform across nutrients or models. Notably, ViT-B-16 excelled in macronutrient prediction, while R101 was more accurate for mass and energy estimation, although differences were lower for the latter target variables. We also demonstrated that data curation, especially ingredient–mass correction, significantly improves model performance, though the magnitude of improvement varies across targets and architectures. Our analysis revealed dish categories that remain systematically mispredicted—even with curated data (i.e., frame filtered out)—highlighting key areas for future research in both modeling and dataset design/curation. These findings offer new insight into how architecture choice and data quality interact in complex nutritional estimation tasks, and they provide a refined baseline for future methods, including contamination with different data sources (e.g., [19,20]). Future work will focus on the following: (1) integrating ingredient classification into the current regression pipeline; (2) exploring 3D volume estimation workflows in Nutrition5k, Nutrition5k360, and other datasets [37,38,60,64,65]; (3) benchmarking predictions against expert assessments and leading food-logging applications [66]; (4) evaluating the selected models on a pilot set of 50 traditional Italian recipes—complete with images and expanded nutritional profiles—currently being compiled.

## Figures and Tables

**Table 1:** Model name abbreviations used in tables and figures.

Original Model Identifier ^1^	Label Used in Tables and Figures ^2^
Inception_V3_IMAGENET1K_V1_2+1	IncV3_2+1
Inception_V3_IMAGENET1K_V1_2+2	IncV3_2+2
ResNet101_IMAGENET1K_V2_2+1	R101_2+1
ResNet101_IMAGENET1K_V2_2+2	R101_2+2
ResNet50_IMAGENET1K_V2_2+1	R50_2+1
ResNet50_IMAGENET1K_V2_2+2	R50_2+2
ViT_B_16_IMAGENET1K_SWAG_E2E_V1_2+1	ViT-B-16_2+1
ViT_B_16_IMAGENET1K_SWAG_E2E_V1_2+2	ViT-B-16_2+2

^1^ Model identifiers for the feature extraction part, as provided by the original model sources (i.e., PyTorch Vision Models [49]), are listed in the left column. ^2^ Abbreviated labels (right column) are used for clarity and brevity throughout the tables and figures in the main text and Supplementary Material.

**Table 2:** Descriptive statistics on observed values for mass, energy, and macronutrient content for dishes included in the test set from Nutrition5k, before or after ingredient–mass correction and matching with Italian-FCDB-specific nutritional values. Nutrition5k test set (*n* = 676).

Single Available Datasets ^1^					Merged Dataset ^1^
Target Variable	US FCDB—No Correction	US FCDB—Correction	IT FCDB—No Correction	IT FCDB—Correction	
**Mass (g)**					
Median (Q1, Q3)	142.0 (70.5, 250.0)	142.0 (70.0, 249.0)	143.0 (70.5, 251.5)	142.0 (70.0, 249.0)	142.0 (70.0, 250.0)
Mean (SD)	189.1 (332.1)	177.3 (142.9)	205.9 (467.1)	177.4 (142.9)	187.4 (304)
Min, Max	1.0, 7974.0	1.0, 871.0	1.0, 8094.0	1.0, 871.0	1.0, 8094.0
**Energy content (kcal)**					
Median (Q1, Q3)	161.9 (61.5, 343.4)	160.0 (61.2, 339.7)	167.2 (60.6, 333.3)	165.6 (60.3, 329.8)	164.5 (60.8, 335.3)
Mean (SD)	252.1 (536.2)	224.0 (205.6)	264.7 (687.8)	227.5 (218.1)	242.1 (461.0)
Min, Max	0.0, 9485.8	0.0, 1050.5	0.0, 12,376.1	0.0, 1332.6	0.0, 12,376.1
**Protein content (g)**					
Median (Q1, Q3)	8.7 (1.6, 23.0)	8.5 (1.6, 22.7)	7.9 (1.6, 22.0)	7.9 (1.6, 21.8)	8.3 (1.6, 22.2)
Mean (SD)	15.7 (19.3)	15.4 (18.9)	15.2 (18.7)	14.9 (17.9)	15.3 (18.7)
Min, Max	0.0, 105.6	0.0, 105.6	0.0, 123.6	0.0, 89.8	0.0, 123.6
**Fat content (g)**					
Median (Q1, Q3)	6.6 (0.6, 17.6)	6.6 (0.6, 17.4)	7.5 (0.4, 17.4)	7.4 (0.4, 17.2)	6.9 (0.5, 17.4)
Mean (SD)	13.6 (48.3)	11.1 (13.5)	15.4 (66.8)	11.9 (15.1)	13.0 (42.4)
Min, Max	0.0, 875.5	0.0, 84.2	0.0, 1221.8	0.0, 115.8	0.0, 1221.8
**Carbohydrates content (g)**					
Median (Q1, Q3)	13.1 (4.4, 25.7)	13.0 (4.4, 25.4)	9.1 (2.3, 20.5)	9.0 (2.2, 20.5)	11.3 (3.3, 23.5)
Mean (SD)	19.2 (31.5)	17.5 (16.4)	15.0 (17.9)	14.6 (16.9)	16.6 (21.7)
Min, Max	0.0, 506.1	0.0, 85.8	0.0, 138.6	0.0, 116.5	0.0, 506.1

^1^ US FCDB—no correction was directly obtained by using the original file from the Nutrition5k project; US FCDB—correction was obtained from US FCDB—no correction by carrying out correction of portion sizes for selected ingredients in dishes; IT FCDB—no correction was obtained by substituting the nutritional composition from the US FCDB with that from the IT FCDB and this included imputing missing-name ingredients, trace values, and missing values for food items in the Italian FCDB; IT FCDB—correction was obtained after correcting portion sizes for selected ingredients in dishes. The merged dataset contained all the dishes from the four available datasets and therefore showed repeated dish IDs. Abbreviations: FCDB, Food Composition Database.

## Data Availability

The current analysis was based on a publicly archived dataset available at: https://github.com/google-research-datasets/Nutrition5k (accessed on 20 June 2025). We derived from this dataset three additional datasets, which are not readily available because they are part of an ongoing study. Requests to access the datasets should be directed to the corresponding author’s email. Codes used to perform the described statistical analyses are available upon request at the corresponding author’s email.

## Supplementary Materials

The following supporting information can be downloaded at: https://www.mdpi.com/article/10.3390/nu17132196/s1. Table S1: Descriptive statistics on predicted values for mass, energy, and macronutrients content of dishes before and after ingredient–mass correction and matching with either US- or Italian-Food Composition Database-specific nutritional values for dishes in the test set from the Nutrition5k dataset. Information was separately provided by dataset and algorithm. Table S2: Percentages of perfect agreement obtained by cross-classifying dishes based on quartiles of observed and predicted values. Table S3: Unweighted Cohen’s kappa coefficients obtained by cross-classifying dishes based on quartiles of observed and predicted values. Table S4: Performance metrics for single target variables, algorithms, and available datasets, as well as median metrics across datasets, before frame filtering. Table S5: List of incorrectly predicted dishes across target variables and datasets, as grouped by similarity in ingredient content. Table S6: Performance metrics for single target variables, algorithms, and available datasets, as well as median metrics across datasets, after frame filtering. Table S7: Sensitivity analysis: percentages of perfect agreement of pairs of predicted and computed energy contents under the 4-task and 5-task deep learning algorithms, before and after frame filtering. Table S8: Sensitivity analysis: unweighted Cohen’s kappa coefficients of pairs of predicted and computed energy contents under the 4-task and 5-task deep learning algorithms, before and after frame filtering. Table S9: Sensitivity analysis: performance metrics for predicted and computed energy contents under the 4-task and 5-task deep learning algorithms, before and after frame filtering. Figure S1: Examples of food images; observed and predicted mass and nutritional composition for the different combinations of algorithms and available datasets. Figure S2: Representative training and validation loss curves supporting the choice of fixed training epochs. Figures S3–S7: Bland–Altman plots representing the raw absolute difference between predicted and observed values on the test set (*y*-axis) versus the mean of the predicted and observed values for each target variable (*x*-axis), with corresponding 95% limits of agreement.

## Abbreviations

The following abbreviations are used in this manuscript:

AI	Artificial Intelligence
DL	Deep Learning
FCDB	Food Composition DataBase
Inc	Inception
MAE	Mean Absolute Error
MAPE	Mean Absolute Percentage Error
MLP	Multi-Layer Perceptron
ReLU	Rectified Linear Unit
RGB	Red, Green, and Blue
ResNet	Residual Network
RMSE	Root Mean Squared Error
ViT	Vision Transformer
