# Supplementary material for: 2D Prediction of the Nutritional Composition of Dishes from Food Images: Deep Learning Algorithm Selection and Data Curation Beyond the Nutrition5k Project
Source: Nutrients. 2025 Jun 30;17(13):2196. doi: 10.3390/nu17132196 (PMC12252204; doi:10.3390/nu17132196)
Supplement: Supplementary file 1 [file nutrients-17-02196-s001.zip › nutrients-3658964-supplementary.pdf]

2D Prediction of the Nutritional Composition of Dishes from Food Images: Deep Learning Algorithm Selection and Data Curation beyond the Nutrition5k Project

Bianco et al.

Supplemental methods

Detailed description of deep learning algorithms implemented in the current analyses

Residual Network

Residual Networks (ResNets) represent a class of deep neural networks primarily composed of convolutional layers. A ResNet is built by stacking multiple residual blocks, each containing several fundamental components, including convolutional layers for feature extraction, nonlinear functions to introduce complexity, pooling operations to reduce spatial dimensions while preserving critical information, and batch normalization to stabilize training by normalizing activations at each layer. As the input image passes through the network, its spatial dimensions progressively decrease, while the number of feature (channels) increases. This trade-off enables the network to capture higher-level semantic representations (e.g., objects) in deeper layers, by building upon lower-level features (e.g., edges or corners) extracted at earlier layers. A distinguishing feature of ResNet is the incorporation of "skip connections" (i.e., shortcuts connections), which allow the input to bypass one or more layers and be directly added to the output. This design prevents the problem of vanishing gradient, a common issue in deep networks, where gradients diminish as they propagate through multiple layers, thereby facilitating more effective gradient flow and improving training stability in very deep models.

Inception Network

The Inception Network is a deep convolutional neural network characterized by its novel inception module, which simultaneously applies multiple convolutional filters of varying sizes (e.g., 1×1, 3×3, and 5×5) to the same input. This architectural design enables the network to extract features at multiple spatial scales in parallel, allowing it to capture fine-grained details as well as broader patterns from the same input and facilitating a more comprehensive feature extraction process. The outputs of these parallel convolutional operations are then combined, allowing the network to integrate diverse spatial information at each layer. Similar to ResNets, the Inception Network progressively reduces the spatial dimensions of the input while increasing the number of feature channels as depth increases. To enhance computational efficiency, the authors perform spatial factorization of the convolutional filters, both resulting in smaller convolutions (e.g., 1x1) and asymmetric convolutions (e.g., nx1 and 1xn), to reduce the number of parameters before applying larger convolutional filters, thereby minimizing the overall computational cost. Additionally, auxiliary classifiers (i.e., smaller prediction layers positioned at intermediate depths) are incorporated to mitigate the vanishing gradient problem by providing supplementary learning signals during training, thereby improving convergence and stability in deep architectures.

Vision Transformer

Vision Transformers (ViTs) represent a paradigm shift in image processing by diverging from conventional convolutional neural networks. Rather than processing an image as a whole and sliding through it with locally applied filters, ViTs divide the input image into fixed-size patches (e.g., 16x16 pixels), each of which is subsequently flattened into a vector and encoded via a linear transformation. This approach enables the sequence of patches to be interpreted as a series of tokens, analogous to word embeddings in natural language processing, allowing the model to leverage the Transformer architecture—originally developed for language modeling—to analyze visual data. Since this method discards the natural spatial structure of the image,

positional encodings are introduced to retain information about the location of each patch, functioning similarly to spatial coordinates. The Transformer encoder uses self-attention mechanisms to establish relationships among all patches, allowing thereby capturing global dependencies within the image. Unlike convolutional neural networks, which progressively build hierarchical feature representations through local receptive fields, ViTs can model long-range dependencies from the initial layers, facilitating a more holistic understanding of the image content. To enhance stability during training, ViTs incorporate skip connections similar to those used in ResNet architectures. However, in contrast to convolutional neural networks, ViTs do not inherently encode spatial locality and translational equivariance, necessitating the use of large-scale datasets to achieve optimal performance. The absence of these inductive biases, which convolutional neural networks naturally exploit (e.g., the assumption that spatially proximate pixels are more relevant than distant ones), renders ViTs highly effective for large datasets but comparatively less efficient when trained on smaller datasets without extensive pretraining.

Supplemental results

Table S1. Descriptive statistics on predicted values for mass, energy, and macronutrients content of dishes before and after ingredient-mass correction and matching with either US- or Italian-Food Composition Database-specific nutritional values for dishes in the test set from the Nutrition5k dataset. Information was separately provided by dataset and algorithm. Nutrition5k test set (n = 676).

Target variable, dataset[1], and algorithm	Minimum	Q1	Median	Q3	Maximum	Mean	SD
Mass (g)							
**US FCDB—no correction**							
IncV3_2+1	11.989	78.460	149.029	243.804	667.309	175.567	119.070
IncV3_2+2	8.345	82.692	154.692	257.083	920.139	185.216	131.994
R101_2+1	10.483	77.415	154.325	260.761	804.408	182.848	131.531
R101_2+2	12.549	80.182	155.870	264.914	811.699	186.869	132.116
R50_2+1	10.995	76.166	153.896	259.977	794.653	183.216	131.574
R50_2+2	12.439	79.555	155.980	258.996	812.958	184.906	132.786
ViT-B-16_2+1	8.600	77.167	148.562	246.794	811.833	176.981	122.878
ViT-B-16_2+2	8.345	82.692	154.692	257.083	920.139	185.216	131.994
**US FCDB—correction**							
IncV3_2+1	13.209	76.956	150.509	248.492	700.395	176.779	122.460
IncV3_2+2	13.676	76.691	146.318	245.957	773.388	176.409	123.264
R101_2+1	11.898	74.558	151.948	254.598	810.591	179.795	129.482
R101_2+2	11.175	76.655	152.266	251.004	811.542	179.710	128.035
R50_2+1	10.065	77.313	151.532	246.361	794.116	178.766	126.415
R50_2+2	10.110	78.152	151.895	249.858	767.742	179.521	127.690
ViT-B-16_2+1	10.736	77.782	146.475	245.508	827.270	174.745	121.122
ViT-B-16_2+2	8.499	76.318	148.641	254.667	805.071	177.517	124.557
**IT FCDB—no correction**							
IncV3_2+1	14.362	78.523	151.278	252.297	840.150	179.400	127.828
IncV3_2+2	16.683	81.294	151.601	248.388	774.850	179.322	124.699
R101_2+1	12.251	76.848	155.851	251.390	811.516	179.98	127.058
R101_2+2	11.778	78.182	156.242	256.914	858.812	183.928	131.479
R50_2+1	13.519	79.803	155.035	257.478	775.501	180.828	125.393

Target variable, dataset[1], and algorithm	Minimum	Q1	Median	Q3	Maximum	Mean	SD
R50_2+2	14.628	77.307	151.389	255.324	831.289	180.053	130.101
ViT-B-16_2+1	9.468	77.178	148.882	247.837	858.081	176.779	124.204
ViT-B-16_2+2	9.601	76.350	145.187	244.340	803.312	174.455	122.162
**IT FCDB—correction**							
IncV3_2+1	12.741	76.896	149.564	246.829	846.107	177.086	126.658
IncV3_2+2	14.563	76.606	144.564	239.602	705.234	172.109	118.026
R101_2+1	12.973	78.508	158.148	258.043	784.960	182.376	128.297
R101_2+2	12.464	76.302	153.909	249.132	832.611	177.936	126.631
R50_2+1	12.340	75.194	149.358	243.864	818.391	175.475	124.730
R50_2+2	12.476	73.248	147.020	244.284	794.214	173.613	123.567
ViT-B-16_2+1	9.903	76.584	146.949	243.318	828.733	173.389	120.967
ViT-B-16_2+2	10.333	74.397	143.945	247.973	802.560	173.938	123.074
Energy content (kcal)							
**US FCDB—no correction**							
IncV3_2+1	4.749	73.189	171.423	318.103	839.947	211.837	164.620
IncV3_2+2	2.450	71.426	179.457	324.222	782.837	217.700	169.879
R101_2+1	3.061	74.806	176.549	338.438	957.235	223.536	178.885
R101_2+2	2.727	73.597	176.066	328.564	847.582	219.088	173.332
R50_2+1	3.682	70.818	174.440	334.196	903.971	220.078	176.532
R50_2+2	3.809	69.867	170.604	329.921	919.872	217.213	175.830
ViT-B-16_2+1	1.582	73.160	177.642	317.225	797.581	211.354	162.167
ViT-B-16_2+2	2.450	71.426	179.457	324.222	782.837	217.700	169.879
**US FCDB—correction**							
IncV3_2+1	4.486	72.799	174.193	312.210	806.510	211.116	161.165
IncV3_2+2	5.012	70.902	172.772	325.804	866.676	216.743	169.466
R101_2+1	3.076	67.133	165.719	324.468	897.445	215.453	176.270
R101_2+2	4.326	71.626	172.578	324.581	901.615	216.787	173.480
R50_2+1	3.795	68.516	169.911	323.592	893.864	213.064	170.517
R50_2+2	3.174	72.027	172.450	328.393	903.872	217.691	174.358

Target variable, dataset[1], and algorithm	Minimum	Q1	Median	Q3	Maximum	Mean	SD
ViT-B-16_2+1	2.962	71.442	175.015	313.451	778.592	206.272	158.485
ViT-B-16_2+2	2.510	72.592	178.562	321.872	788.018	210.252	161.242
**IT FCDB—no correction**							
IncV3_2+1	6.000	72.641	187.025	328.627	801.535	222.230	175.832
IncV3_2+2	6.456	73.068	186.211	325.874	866.069	220.955	175.866
R101_2+1	4.081	72.029	185.674	327.314	925.241	223.643	180.005
R101_2+2	3.254	76.821	190.447	336.735	928.852	230.404	183.906
R50_2+1	6.110	76.244	184.142	325.792	957.968	223.189	178.599
R50_2+2	4.434	71.126	183.998	339.073	982.392	227.805	188.426
ViT-B-16_2+1	2.277	75.039	184.466	315.538	930.568	213.937	167.283
ViT-B-16_2+2	3.123	72.620	188.120	315.731	942.894	215.169	169.504
**IT FCDB—correction**							
IncV3_2+1	5.259	69.017	167.787	308.190	850.381	208.964	168.689
IncV3_2+2	5.918	71.143	172.486	321.870	834.231	214.939	171.573
R101_2+1	3.873	71.336	181.510	320.481	951.214	218.884	177.677
R101_2+2	4.869	67.796	165.120	310.273	972.667	208.651	172.866
R50_2+1	5.991	70.739	178.455	325.476	952.355	220.980	180.643
R50_2+2	3.684	71.431	192.502	328.854	942.238	225.362	181.261
ViT-B-16_2+1	2.787	73.943	182.214	308.889	940.190	212.008	168.041
ViT-B-16_2+2	3.564	71.106	179.319	303.868	941.397	208.321	165.006
Protein content (g)							
**US FCDB—no correction**							
IncV3_2+1	0.258	2.682	9.153	20.197	57.919	13.204	12.720
IncV3_2+2	-1.761	1.439	8.598	19.083	60.461	12.620	13.405
R101_2+1	0.225	2.462	10.519	24.047	76.708	15.351	15.861
R101_2+2	0.166	2.366	9.821	21.938	71.668	14.274	14.557
R50_2+1	0.111	2.314	10.113	23.206	75.478	15.000	15.620
R50_2+2	0.305	2.286	9.284	22.069	76.524	14.299	15.046
ViT-B-16_2+1	0.184	1.931	8.922	20.276	77.313	13.234	13.750

Target variable, dataset[1], and algorithm	Minimum	Q1	Median	Q3	Maximum	Mean	SD
ViT-B-16_2+2	-1.761	1.439	8.598	19.083	60.461	12.620	13.405
**US FCDB—correction**							
IncV3_2+1	-0.542	2.172	8.594	19.062	54.704	12.083	11.961
IncV3_2+2	0.011	2.659	9.390	21.970	55.664	13.995	13.264
R101_2+1	0.135	1.887	9.047	21.284	73.106	13.829	14.772
R101_2+2	0.269	2.198	9.611	21.483	73.009	14.141	14.665
R50_2+1	0.251	2.343	9.967	22.711	67.562	14.624	14.965
R50_2+2	0.169	2.142	9.821	22.430	75.023	14.392	15.021
ViT-B-16_2+1	0.090	2.070	8.921	20.743	75.446	13.450	14.024
ViT-B-16_2+2	0.222	2.091	9.577	22.418	80.511	14.186	14.641
**IT FCDB—no correction**							
IncV3_2+1	0.251	2.826	9.737	20.065	60.003	13.462	12.914
IncV3_2+2	-0.264	3.184	10.562	20.955	50.125	13.425	11.94
R101_2+1	0.281	2.310	10.213	20.878	62.977	13.979	14.003
R101_2+2	0.239	2.839	10.369	22.484	62.131	14.719	14.125
R50_2+1	0.022	2.016	9.084	19.959	59.613	13.014	13.315
R50_2+2	0.289	2.422	10.036	21.301	62.323	14.017	14.035
ViT-B-16_2+1	0.212	2.685	10.108	21.260	75.095	14.237	14.2
ViT-B-16_2+2	0.254	2.223	9.481	19.968	76.143	13.356	13.566
**IT FCDB—correction**							
IncV3_2+1	-0.824	1.871	7.720	17.582	57.707	11.474	11.703
IncV3_2+2	-0.211	2.892	9.748	19.928	47.937	12.886	11.590
R101_2+1	0.296	2.919	11.678	23.584	67.515	15.706	15.091
R101_2+2	0.204	2.320	8.961	19.170	52.351	12.502	12.020
R50_2+1	0.266	2.418	9.993	21.084	63.177	14.189	14.135
R50_2+2	0.199	2.435	10.409	21.577	62.285	14.155	13.960
ViT-B-16_2+1	0.188	2.448	9.984	19.953	70.286	13.563	13.537
ViT-B-16_2+2	0.250	2.519	9.970	21.141	72.847	14.020	14.201

Fat content (g)

Target variable, dataset[1], and algorithm	Minimum	Q1	Median	Q3	Maximum	Mean	SD
**US FCDB—no correction**							
IncV3_2+1	0.049	2.306	8.087	17.772	64.362	11.025	10.405
IncV3_2+2	-0.316	2.127	8.464	17.465	61.856	11.163	10.671
R101_2+1	-0.037	2.254	8.577	18.486	81.061	11.815	11.971
R101_2+2	-0.021	2.226	7.997	17.183	65.257	10.751	10.372
R50_2+1	-0.046	2.225	8.398	17.988	73.499	11.425	11.278
R50_2+2	0.127	1.887	7.581	17.136	71.026	10.710	10.764
ViT-B-16_2+1	-0.075	1.963	7.690	16.291	50.987	10.390	10.030
ViT-B-16_2+2	-0.316	2.127	8.464	17.465	61.856	11.163	10.671
**US FCDB—correction**							
IncV3_2+1	0.032	2.226	7.328	16.262	56.520	10.076	9.151
IncV3_2+2	0.082	1.591	7.144	16.571	60.302	10.206	9.970
R101_2+1	-0.188	1.414	6.810	15.987	69.566	10.013	10.535
R101_2+2	0.050	1.773	8.092	17.650	71.250	11.083	11.050
R50_2+1	-0.051	1.897	7.676	16.661	69.234	10.574	10.637
R50_2+2	0.032	1.673	7.768	17.266	70.341	10.820	10.966
ViT-B-16_2+1	-0.244	1.721	7.343	16.194	53.147	10.191	10.103
ViT-B-16_2+2	0.032	1.948	7.992	16.605	54.339	10.493	10.311
**IT FCDB—no correction**							
IncV3_2+1	0.047	2.782	9.540	19.400	67.807	12.307	11.427
IncV3_2+2	0.075	2.163	8.314	17.672	64.835	11.248	10.952
R101_2+1	0.002	2.231	9.313	19.399	81.143	12.613	12.651
R101_2+2	-0.071	1.830	8.588	18.269	74.048	11.566	11.589
R50_2+1	-0.243	1.709	8.178	17.724	78.881	11.422	11.749
R50_2+2	0.023	1.958	8.503	19.241	82.211	12.308	12.763
ViT-B-16_2+1	-0.638	2.028	8.455	16.814	75.945	11.207	11.414
ViT-B-16_2+2	0.040	1.822	8.689	17.461	74.336	11.537	11.508
**IT FCDB—correction**							
IncV3_2+1	-0.905	1.257	6.077	15.345	70.096	9.478	10.460
IncV3_2+2	0.072	2.012	7.759	17.470	62.927	11.084	10.735

Target variable, dataset[1], and algorithm	Minimum	Q1	Median	Q3	Maximum	Mean	SD
R101_2+1	-0.172	1.869	8.416	18.138	83.503	11.889	12.384
R101_2+2	0.025	1.660	7.673	17.014	74.644	10.921	11.180
R50_2+1	-0.273	1.736	8.247	17.877	78.931	11.599	12.093
R50_2+2	0.018	2.037	8.955	18.768	79.813	12.257	12.368
ViT-B-16_2+1	0.183	2.724	9.055	17.764	79.600	12.018	11.571
ViT-B-16_2+2	0.010	1.740	8.033	16.086	74.283	10.653	10.715
Carbohydrates content (g)							
**US FCDB—no correction**							
IncV3_2+1	0.183	7.859	15.551	23.424	51.955	16.474	10.050
IncV3_2+2	-0.543	7.963	17.041	25.075	57.654	17.998	11.801
R101_2+1	0.627	7.004	15.583	25.650	61.715	17.784	12.678
R101_2+2	0.216	6.801	15.178	24.537	61.924	17.313	12.375
R50_2+1	0.164	6.475	14.396	24.164	56.764	16.642	12.069
R50_2+2	0.472	7.039	15.238	25.068	60.413	17.330	12.259
ViT-B-16_2+1	-0.024	6.241	13.419	22.832	53.762	15.614	11.624
ViT-B-16_2+2	-0.543	7.963	17.041	25.075	57.654	17.998	11.801
**US FCDB—correction**							
IncV3_2+1	0.462	7.348	13.614	20.555	45.103	14.584	8.883
IncV3_2+2	0.969	8.176	15.331	21.614	48.728	15.633	8.859
R101_2+1	0.162	6.419	14.234	24.158	62.601	16.665	12.219
R101_2+2	0.166	6.158	13.933	22.480	56.584	15.897	11.546
R50_2+1	0.226	6.508	14.732	23.882	57.620	16.795	12.020
R50_2+2	0.349	6.607	14.294	23.429	58.718	16.403	11.776
ViT-B-16_2+1	-0.181	6.409	13.789	22.836	58.202	15.974	11.840
ViT-B-16_2+2	0.013	6.381	14.040	23.301	56.766	16.202	12.066
**IT FCDB—no correction**							
IncV3_2+1	-0.154	4.904	12.230	19.168	49.066	13.106	9.338
IncV3_2+2	0.855	4.806	10.629	16.534	46.382	11.683	8.187
R101_2+1	-1.306	3.367	10.326	18.651	55.249	12.500	10.788

Target variable, dataset[1], and algorithm	Minimum	Q1	Median	Q3	Maximum	Mean	SD
R101_2+2	-0.023	3.995	10.654	19.133	55.214	12.872	10.463
R50_2+1	-0.138	3.876	10.970	19.902	53.981	13.240	10.790
R50_2+2	-0.034	3.620	10.551	19.574	54.379	12.986	11.029
ViT-B-16_2+1	-0.387	4.058	10.698	19.386	58.382	13.263	11.203
ViT-B-16_2+2	0.040	3.738	10.825	19.674	59.867	13.311	11.299
**IT FCDB—correction**							
IncV3_2+1	-1.257	4.006	10.534	16.942	41.867	11.260	8.240
IncV3_2+2	0.595	4.365	9.778	15.774	42.196	11.086	7.861
R101_2+1	0.101	4.222	11.726	20.884	62.814	14.046	11.484
R101_2+2	1.132	4.353	10.209	17.604	45.489	12.060	8.928
R50_2+1	-2.055	2.800	9.419	18.342	52.611	11.625	10.148
R50_2+2	0.076	3.238	9.819	18.717	55.693	12.294	10.592
ViT-B-16_2+1	0.069	4.059	10.457	19.231	56.605	13.148	11.080
ViT-B-16_2+2	-0.150	3.367	9.541	18.818	61.622	12.570	10.973

[1]US FCDB—no correction was directly obtained by using the original file from the Nutrition5k project; US FCDB—correction was obtained from US FCDB—no correction by carrying out correction of portion sizes for selected ingredients in dishes; IT FCDB—no correction was obtained by substituting the nutritional composition from the US FCDB with that from the IT FCDB and this included imputing missing-name ingredients, trace values, and missing values for food items in the Italian FCDB; IT FCDB—correction was obtained after correcting portion sizes for selected ingredients in dishes. Abbreviations: FCDB, Food Composition Database

Tables S2. Percentages of perfect agreement obtained by cross-classifying dishes based on quartiles of observed and predicted values. Nutrition5k test set (*n* = 676).

Target variable and dataset[1]	IncV3_2+1	IncV3_2+2	R101_2+1	R101_2+2	R50_2+1	R50_2+2	ViT-B-16_2+1	ViT-B-16_2+2
Mass (g)								
US FCDB—no correction	0.786	0.803	0.808	0.808	0.806	0.811	0.808	0.803
US FCDB—correction	0.802	0.778	0.817	0.821	0.817	0.805	0.817	0.822
IT FCDB—no correction	0.788	**0.766**	0.814	0.817	0.814	0.821	0.815	0.809
IT FCDB—correction	0.800	0.796	0.809	0.822	0.817	0.811	**0.827**	0.812
Energy content (kcal)								
US FCDB—no correction	0.778	0.771	0.803	0.800	0.780	0.806	**0.818**	0.771
US FCDB—correction	0.784	0.778	0.794	0.806	0.809	0.805	0.814	0.811
IT FCDB—no correction	**0.756**	0.756	0.783	0.787	0.788	0.788	0.781	0.763
IT FCDB—correction	0.774	0.778	0.799	0.787	0.791	0.791	0.794	0.787
Protein content (g)								
US FCDB—no correction	0.672	0.716	0.765	0.746	0.759	0.763	0.778	0.716
US FCDB—correction	0.691	0.641	0.775	0.763	0.769	0.759	0.771	0.766
IT FCDB—no correction	0.680	**0.624**	0.784	0.768	0.763	0.772	0.769	0.772
IT FCDB—correction	0.703	0.648	0.769	0.756	0.759	0.768	**0.783**	0.771
Fat content (g)								
US FCDB—no correction	**0.651**	0.678	0.719	0.712	0.712	0.714	0.753	0.678
US FCDB—correction	0.675	0.654	0.725	0.735	0.738	0.732	0.763	0.754
IT FCDB—no correction	0.669	0.655	0.735	0.716	0.744	0.735	0.777	0.780
IT FCDB—correction	0.683	0.660	0.743	0.714	0.744	0.731	0.757	**0.783**
Carbohydrates content (g)								
US FCDB—no correction	0.525	0.572	0.691	0.666	0.666	0.670	0.720	0.572
US FCDB—correction	0.525	**0.491**	0.697	0.704	0.683	0.683	0.722	0.728
IT FCDB—no correction	0.562	**0.491**	0.740	0.683	0.701	0.700	0.759	0.760
IT FCDB—correction	0.541	0.506	0.706	0.612	0.706	0.720	0.762	**0.780**

[1]US FCDB—no correction was directly obtained by using the original file from the Nutrition5k project; US FCDB—correction was obtained from US FCDB—no correction by carrying out correction of portion sizes for selected ingredients in dishes; IT FCDB—no correction was obtained by substituting the nutritional composition from the US FCDB with that from the IT FCDB and this included imputing missing-name ingredients, trace values, and missing values for food items in the Italian FCDB; IT FCDB—correction was obtained after correcting portion sizes for selected ingredients in dishes. Values in bold typeface with a light blue cell background indicate the minimum and maximum percentages of perfect agreement for each target variable. Abbreviations: FCDB, Food Composition Database

Tables S3. Unweighted Cohen's kappa coefficients obtained by cross-classifying dishes based on quartiles of observed and predicted values. Nutrition5k test set (*n* = 676).

Target variable and dataset[1]	IncV3_2+1	IncV3_2+2	R101_ 2+1	R101_ 2+2	R50_2+1	R50_2+2	ViT-B-16_2+1	ViT-B-16_2+2
Mass (g)								
US FCDB—no correction	0.714*[2]	0.738*	0.744*	0.744*	0.742*	0.748*	0.744*	0.738*
US FCDB—correction	0.736*	0.704*	0.755*	0.761*	0.755*	0.740*	0.755*	0.763*
IT FCDB—no correction	0.718*	**0.688***	0.751*	0.755*	0.751*	0.761*	0.753*	0.746*
IT FCDB—correction	0.734*	0.728*	0.746*	0.763*	0.755*	0.748*	**0.769***	0.750*
Energy content (kcal)								
US FCDB—no correction	0.704*	0.694*	0.738*	0.734*	0.706*	0.742*	**0.757***	0.694*
US FCDB—correction	0.712*	0.704*	0.726*	0.742*	0.746*	0.740*	0.751*	0.748*
IT FCDB—no correction	0.675*	**0.675***	0.710*	0.716*	0.718*	0.718*	0.708*	0.684*
IT FCDB—correction	0.698*	0.704*	0.732*	0.716*	0.722*	0.722*	0.726*	0.716*
Protein content (g)								
US FCDB—no correction	0.562*	0.621*	0.686*	0.661*	0.679*	0.684*	0.704*	0.621*
US FCDB—correction	0.588*	0.521*	0.700*	0.684*	0.692*	0.679*	0.694*	0.688*
IT FCDB—no correction	0.574*	**0.499***	0.712*	0.690*	0.684*	0.696*	0.692*	0.696*
IT FCDB—correction	0.604*	0.531*	0.692*	0.675*	0.679*	0.690*	**0.710***	0.694*
Fat content (g)								
US FCDB—no correction	**0.535***	0.570*	0.625*	0.615*	0.615*	0.619*	0.671*	0.570*
US FCDB—correction	0.566*	0.538*	0.633*	0.647*	0.651*	0.643*	0.684*	0.673*
IT FCDB—no correction	0.558*	0.540*	0.647*	0.621*	0.659*	0.647*	0.702*	0.706*
IT FCDB—correction	0.578*	0.546*	0.657*	0.619*	0.659*	0.641*	0.677*	**0.710***
Carbohydrates content (g)								
US FCDB—no correction	0.367*	0.430*	0.588*	0.554*	0.554*	0.560*	0.627*	0.430*
US FCDB—correction	0.367*	**0.321***	0.596*	0.606*	0.578*	0.578*	0.629*	0.637*
IT FCDB—no correction	0.416*	**0.321***	0.653*	0.578*	0.602*	0.600*	0.679*	0.680*
IT FCDB—correction	0.389*	0.341*	0.607*	0.483*	0.607*	0.627*	0.682*	**0.706***

[1]US FCDB—no correction was directly obtained by using the original file from the Nutrition5k project; US FCDB—correction was obtained from US FCDB—no correction by carrying out correction of portion sizes for selected ingredients in dishes; IT FCDB—no correction was obtained by substituting the nutritional composition from the US FCDB with that from the IT FCDB and this included imputing missing-name ingredients, trace values, and missing values for food items in the Italian FCDB; IT FCDB—correction was obtained after correcting portion sizes for selected ingredients in dishes. Values in bold typeface with a light blue cell background indicate the minimum and maximum Cohen's kappa

coefficients for each target variable. [2]The asterisk indicates that the corresponding Cohen's kappa coefficient was different from zero at the 0.05 level. Abbreviations: FCDB, Food Composition Database

Table S4. Performance metrics for single target variables, algorithms, and available datasets, as well as median metrics across datasets before frame filtering. Nutrition5k test set (*n* = 676).

Dataset[1] and algorithm	Mass (g)			Energy content (kcal)			Protein content (g)			Fat content (g)			Carbohydrates content (g)		
	RMSE	MAE	MAPE	RMSE	MAE	MAPE	RMSE	MAE	MAPE	RMSE	MAE	MAPE	RMSE	MAE	MAPE
**US FCDB—no correction**															
IncV3_2+1	310.54	47.64	25.19	505.73	80.84	32.07	12.12	7.04	44.85	46.99	7.04	51.79	29.64	9.48	49.49
IncV3_2+2	309.16	44.31	23.43	503.79	78.23	31.03	11.28	6.20	39.50	46.69	6.68	49.10	29.30	8.57	44.75
R101_2+1	309.34	**43.45**	**22.97**	**502.78**	**75.45**	**29.93**	**9.70**	**5.28**	**33.60**	**46.57**	**6.24**	**45.87**	28.88	7.47	38.99
R101_2+2	309.51	45.13	23.86	503.06	75.88	30.10	10.20	5.58	35.53	46.66	6.35	46.65	28.92	7.45	38.89
R50_2+1	309.48	44.84	23.71	503.49	76.87	30.49	10.22	5.73	36.47	46.65	6.50	47.75	28.91	7.31	38.18
R50_2+2	309.63	45.26	23.93	503.53	77.08	30.57	10.49	5.60	35.63	46.68	6.27	46.13	**28.83**	7.25	37.88
ViT-B_2+1	**309.13**	44.17	23.35	504.08	78.20	31.02	10.23	5.39	34.34	46.68	6.27	46.06	28.84	**6.83**	**35.67**
ViT-B_2+2	309.16	44.31	23.43	503.79	78.23	31.03	11.28	6.20	39.50	46.69	6.68	49.10	29.30	8.57	44.75
**US FCDB—correction**															
IncV3_2+1	56.28	35.36	19.95	88.99	54.92	24.52	11.64	6.90	44.76	7.96	5.02	45.34	12.18	8.03	45.97
IncV3_2+2	55.41	35.91	20.25	84.35	53.51	23.89	11.49	7.15	46.38	7.43	4.54	41.02	12.53	8.63	49.39
R101_2+1	48.83	30.52	17.22	77.62	46.65	20.82	9.11	5.19	33.69	6.72	3.96	35.73	8.66	5.47	31.29
R101_2+2	**48.47**	**30.36**	**17.12**	**76.99**	**46.44**	**20.73**	9.16	5.09	33.06	6.30	3.69	33.35	8.96	5.53	31.66
R50_2+1	49.92	31.15	17.57	80.90	47.91	21.39	9.44	5.39	34.96	6.41	3.75	33.90	9.03	5.73	32.80
R50_2+2	50.43	31.46	17.75	77.53	47.48	21.20	9.23	5.14	33.33	**6.29**	3.67	33.10	8.98	5.60	32.04
ViT-B_2+1	51.10	32.89	18.55	87.33	51.07	22.80	9.18	5.16	33.52	6.74	3.88	34.99	8.52	5.21	29.82
ViT-B_2+2	50.22	32.13	18.12	86.76	51.42	22.95	**8.74**	**4.80**	**31.12**	6.51	**3.63**	**32.80**	**8.30**	**4.97**	**28.42**
**IT FCDB—no correction**															
IncV3_2+1	453.01	64.15	31.15	660.97	96.65	36.52	11.40	6.51	42.78	65.36	8.49	54.97	14.07	7.97	52.98
IncV3_2+2	452.77	64.14	31.15	660.75	96.07	36.30	12.40	7.36	48.33	65.57	8.41	54.44	15.54	9.29	61.74
R101_2+1	451.84	60.64	29.45	658.11	**89.52**	**33.82**	10.04	5.23	34.35	65.10	7.88	51.06	11.91	5.71	37.96
R101_2+2	**451.29**	60.27	29.27	**657.65**	90.90	34.34	10.13	5.44	35.71	65.14	7.65	49.54	12.47	6.35	42.20
R50_2+1	452.14	61.63	29.93	658.77	90.11	34.04	10.35	5.22	34.29	65.18	7.53	48.75	12.14	6.16	40.97
R50_2+2	452.17	61.83	30.02	657.77	89.64	33.87	10.19	5.21	34.20	**65.04**	7.55	48.88	11.94	5.98	39.74
ViT-B_2+1	451.36	**60.00**	**29.14**	658.79	90.86	34.33	**9.79**	5.03	33.02	65.13	7.41	47.99	11.07	5.16	34.34
ViT-B_2+2	451.79	60.52	29.39	658.66	90.72	34.27	9.83	**4.80**	**31.51**	65.10	**7.28**	**47.15**	**10.91**	**5.05**	**33.56**

Dataset[1] and algorithm	Mass (g)			Energy content (kcal)			Protein content (g)			Fat content (g)			Carbohydrates content (g)		
	RMSE	MAE	MAPE	RMSE	MAE	MAPE	RMSE	MAE	MAPE	RMSE	MAE	MAPE	RMSE	MAE	MAPE
**IT FCDB—correction**															
IncV3_2+1	54.09	34.54	19.46	99.69	57.54	25.30	10.56	6.28	42.29	8.92	4.98	41.82	13.52	7.98	54.48
IncV3_2+2	56.30	35.40	19.95	98.52	57.56	25.30	11.33	6.96	46.85	8.75	4.95	41.56	14.24	8.80	60.14
R101_2+1	51.11	31.94	18.00	**89.95**	51.87	22.80	8.28	5.03	33.82	7.29	3.90	32.76	10.08	5.56	37.96
R101_2+2	**48.86**	**30.22**	**17.03**	93.59	**51.61**	**22.69**	9.66	5.49	36.93	8.29	4.35	36.60	12.06	7.17	48.94
R50_2+1	50.02	30.97	17.45	91.01	52.53	23.09	8.70	5.13	34.52	7.54	4.08	34.28	11.19	5.87	40.09
R50_2+2	51.20	31.70	17.87	91.33	53.18	23.38	8.35	4.77	32.08	7.41	3.97	33.37	10.61	5.45	37.22
ViT-B_2+1	51.12	32.12	18.10	91.38	52.80	23.21	8.18	4.80	32.32	**6.95**	4.05	34.08	9.51	4.79	32.74
ViT-B_2+2	51.84	32.84	18.51	95.03	54.75	24.07	**7.72**	**4.40**	**29.64**	7.36	**3.85**	**32.38**	**9.49**	**4.64**	**31.68**
**Overall median**															
IncV3_2+1	183.41	41.50	22.57	302.71	69.19	28.68	11.52	6.71	43.77	27.95	6.03	48.56	13.80	8.00	51.23
IncV3_2+2	182.73	40.11	21.84	301.16	67.90	28.17	11.41	7.05	46.61	27.72	5.81	45.33	14.89	8.72	54.76
R101_2+1	180.22	**37.69**	**20.49**	**296.36**	**63.66**	**26.36**	9.40	5.21	33.76	26.93	**5.10**	40.80	11.00	5.63	37.96
R101_2+2	**179.19**	37.74	**20.49**	298.33	63.75	26.39	9.90	5.46	35.62	27.47	5.35	41.63	12.27	6.76	40.55
R50_2+1	179.75	38.00	20.64	297.25	64.70	26.79	9.83	5.30	34.74	27.10	5.29	41.02	11.67	6.01	39.13
R50_2+2	180.42	38.48	20.90	297.43	65.13	26.97	9.71	5.17	33.77	27.04	5.12	**39.75**	11.28	5.79	37.55
ViT-B-16_2+1	180.12	38.53	20.95	297.73	65.50	27.12	9.49	5.10	33.27	**26.82**	5.16	40.52	10.29	5.19	33.54
ViT-B-16_2+2	180.50	38.58	20.97	299.41	66.49	27.55	**9.29**	**4.80**	**31.32**	27.02	5.27	39.97	**10.20**	**5.01**	**32.62**

[1]US FCDB—no correction was directly obtained by using the original file from the Nutrition5k project; US FCDB—correction was obtained from US FCDB-no correction by carrying out correction of portion sizes for selected ingredients in dishes; IT FCDB—no correction was obtained by substituting the nutritional composition from the US FCDB with that from the IT FCDB and this included imputing missing-name ingredients, trace values, and missing values for food items in the Italian FCDB; IT FCDB—correction was obtained after correcting portion sizes for selected ingredients in dishes. Values in bold typeface with a light blue cell background indicate the minimum performance metrics (i.e., the minimum error) for each target variable; similarly values with an orange cell background indicate the maximum performance MAPE (i.e., the maximum error) for each target variable as commented in the text. Abbreviations: FCDB, Food Composition Database

Table S5. List of 68 incorrectly predicted dishes across target variables and datasets, as grouped by similarity in ingredient content. Nutrition5k test set (*n* = 664).

Dish ID	Total original frames	Left frames	Removed frames	Percentage of left frames	Percentage of removed frames	Number of algorithms wrongly predicting the dish	Number of ingredients per dish	List of ingredients per dish
***Salad-based* group** (median number of ingredients per dish: 16, IQR: 11.25–20.5)								
**dish_1560367952**	28	1	27	3.6%	96.4%	8	9	Fish, spinach (raw), pizza, onions, country rice, olive oil, salt, garlic, green onions
**dish_1560367980**	28	2	26	7.1%	92.9%	8	10	Asparagus, salt, onions, country rice, olive oil, green onions, pizza, spinach (raw), fish, garlic
**dish_1560368506**	28	15	13	53.6%	46.4%	7	18	Brown rice, fish, shallots, honeydew melons, broccoli, lemon juice, parsley, pork, vinegar, cherry tomatoes, pepper, garlic, salt, country rice, basil, olive oil, onions, green onions
**dish_1560368570**	28	2	26	7.1%	92.9%	8	24	Honeydew melons, pepper, cherry tomatoes, lettuce, mixed greens, pork, mustard, olive oil, lemon juice, onions, carrot, broccoli, green onions, shallots, arugula, vinegar, brown rice, fish, pumpkin seeds, basil, parsley, country rice, salt, garlic
**dish_1560800988**	24	11	13	45.8%	54.2%	8	9	Pepper, cauliflower, chickpeas, salt, bulgur, olive oil, kale, lemon juice, parsley
**dish_1560801020**	24	12	12	50.0%	50.0%	8	16	Cauliflower, garlic, spinach (raw), parmesan cheese, wheat berry, chickpeas, pepper, chicken breast, cherry tomatoes, thyme, lemon juice, olive oil, parsley, kale, bulgur, salt
**dish_1560801041**	26	10	16	38.5%	61.5%	8	22	Pork, zucchini, arugula, chicken breast, cherry tomatoes, rosemary, lemon juice, thyme, olive oil, onions, wheat berry, parmesan cheese, pepper, spinach (raw), cauliflower, garlic, chickpeas, white rice, salt, bulgur, parsley, kale

Dish ID	Total original frames	Left frames	Removed frames	Percentage of left frames	Percentage of removed frames	Number of algorithms wrongly predicting the dish	Number of ingredients per dish	List of ingredients per dish
**dish_1561577848**	28	19	9	67.9%	32.1%	8	17	Salt, white wine, cream, oregano, arugula, lemon juice, olive oil, pepper, chicken, mushroom, wheat berry, butter, rosemary, thyme, shallots, garlic, basil
**dish_1561577947**	32	9	23	28.1%	71.9%	8	22	Olive oil, basil, arugula, blueberries, vinegar, mustard, walnuts, white wine, salt, rosemary, oregano, cream, mushroom, wheat berry, chard, pepper, chicken, shallots, garlic, butter, lemon juice, thyme
**dish_1561661377**	102	13	89	12.7%	87.3%	8	14	Garlic, chard, brown rice, green beans, chicken, salt, carrot, Caesar salad, parsley, chive, rosemary, basil, lemon juice, olive oil
**dish_1562009934**	28	15	13	53.6%	46.4%	8	16	Quinoa, mixed greens, garlic, parsley, mustard, lemon juice, brown rice, arugula, vinegar, olives, shallots, pears, salt, goat cheese, thyme, olive oil
**dish_1562618000**	28	13	15	46.4%	53.6%	8	16	Vinegar, olive oil, garlic, mustard greens, pepper, salt, radishes, lemon juice, thyme, brown rice, parsley, basil, arugula, chicken thighs, chickpeas, broccoli
**dish_1562790855**	146	21	125	14.4%	85.6%	8	21	Radishes, fish, cherry tomatoes, shallots, onions, mustard, parsley, garlic, basil, salt, pepper, pork, lemon juice, brown rice, country rice, cucumbers, olive oil, vinegar, bell peppers, broccoli, green onions
**dish_1563478751**	32	1	31	3.1%	96.9%	8	12	Cream, mushroom, spinach (raw), olive oil, pasta, beef, wine, garlic, sour cream, onions, thyme, salt
**dish_1563909550**	29	25	4	86.2%	13.8%	8	11	Cucumbers, mustard, mustard greens, olive oil, garlic, green beans, tomatoes, salt, fried rice, vinegar, spinach (raw)

Dish ID	Total original frames	Left frames	Removed frames	Percentage of left frames	Percentage of removed frames	Number of algorithms wrongly predicting the dish	Number of ingredients per dish	List of ingredients per dish
**dish_1563909580**	28	13	15	46.4%	53.6%	8	12	Mustard greens, cucumbers, tomatoes, salt, mustard, garlic, fried rice, cheese pizza, vinegar, spinach (raw), green beans, olive oil
**dish_1565030391**	32	8	24	25.0%	75.0%	8	19	White rice, garlic, orange juice, onions, cucumbers, kale, green onions, lettuce, soy sauce, pepper, cherry tomatoes, beef, vinegar, olive oil, broccoli, lime, salt, chili, mixed greens
**dish_1565123881**	31	14	17	45.2%	54.8%	8	13	Caesar salad, olive oil, onions, cheese pizza, zucchini, spinach (cooked), millet, shallots, bok choy, salt, beef, chayote squash, vinegar
**dish_1565810969**	63	30	33	47.6%	52.4%	7	1	Spinach (raw)
**dish_1565898230**	31	10	21	32.3%	67.7%	7	27	Garlic, bell peppers, beef, cherry tomatoes, brown rice, ginger, olive oil, vinegar, spinach (cooked), lime, shallots, bok choy, salmon, millet, vinaigrette, jalapenos, spinach (raw), green beans, feta cheese, cucumbers, lettuce, cilantro, lemon juice, onions, salt, chili, olives
**dish_1565986761**	343	29	314	8.5%	91.5%	8	22	Cherry tomatoes, fish, chive, ground turkey, cream, chard, onions, lemon juice, parsley, olive oil, shallots, thyme, brown rice, salt, white rice, wheat berry, kale, mushroom, celery, carrot, garlic, eggs
**dish_1566414342**	34	5	29	14.7%	85.3%	7	19	Garlic, pepper, mushroom, oregano, cream, wheat berry, butter, basil, salt, shallots, white wine, chicken, quinoa, arugula, thyme, olive oil, rosemary, lemon juice, roasted potatoes
**dish_1566414412**	32	8	24	25.0%	75.0%	8	27	Shallots, olive oil, garlic, lemon juice, basil, cream, wheat berry, mushroom, arugula, cherry tomatoes, quinoa, chicken, pepper,

Dish ID	Total original frames	Left frames	Removed frames	Percentage of left frames	Percentage of removed frames	Number of algorithms wrongly predicting the dish	Number of ingredients per dish	List of ingredients per dish
								bell peppers, white wine, lettuce, feta cheese, roasted potatoes, onions, butter, thyme, rosemary, cucumbers, olives, oregano, vinaigrette, salt
**dish_1566502573**	31	7	24	22.6%	77.4%	8	28	Chive, bok choy, chicken, white rice, pork, arugula, spinach (raw), brown rice, chard, mustard, olive oil, garlic, cucumbers, sugar, rosemary, pepper, sun dried tomatoes, lemon juice, mustard greens, parsley, vinegar, corn on the cob, basil, onions, carrot, soy sauce, salt, chickpeas
**dish_1566587182**	28	7	21	25.0%	75.0%	8	17	Pepper, millet, avocado, spinach (raw), vinegar, olive oil, onions, bell peppers, salt, garlic, cherry tomatoes, chicken, jalapenos, mustard, celery root, mixed greens, tomatoes
**dish_1566590007**	33	8	25	24.2%	75.8%	8	13	Bell peppers, garlic, tomatoes, salt, onions, olive oil, pizza, pepper, tofu, jalapenos, spinach (raw), chicken, millet
**dish_1566590056**	31	8	23	25.8%	74.2%	8	19	Tomatoes, pepper, millet, vinegar, tofu, jalapenos, mustard, olive oil, bell peppers, cherry tomatoes, chicken, chard, spinach (raw), pizza, cucumbers, onions, garlic, salt, figs
**dish_1566851616**	31	5	26	16.1%	83.9%	8	4	Caesar salad, broccoli, cheese pizza, olive oil
**dish_1568305257**	34	7	27	20.6%	79.4%	7	10	Turkey bacon, salt, bell peppers, mustard, olive oil, cucumbers, vinegar, scrambled eggs, garlic, radishes
**dish_1568401302**	31	12	19	38.7%	61.3%	8	7	Fried rice, salt, squash, carrot, avocado, olive oil, tuna salad
***Eggs-based* group** (median number of ingredients per dish: 5, IQR: 3–6)								
**dish_1561999624**	36	2	34	5.6%	94.4%	8	1	Scrambled eggs

Dish ID	Total original frames	Left frames	Removed frames	Percentage of left frames	Percentage of removed frames	Number of algorithms wrongly predicting the dish	Number of ingredients per dish	List of ingredients per dish
**dish_1561999706**	36	11	25	30.6%	69.4%	8	2	Scrambled eggs, sweet potato
**dish_1562691064**	28	13	15	46.4%	53.6%	8	7	Berries, fried rice, olive oil, pineapple, scrambled eggs, roasted potatoes, broccoli
**dish_1563551220**	29	13	16	44.8%	55.2%	8	9	Cream cheese, berries, greek yogurt, milk, chia seeds, bagels, egg whites, olive oil, sweet potato
**dish_1563984242**	30	12	18	40.0%	60.0%	8	5	Sweet potato, egg whites, pineapple, olive oil, cantaloupe
**dish_1563984296**	29	12	17	41.4%	58.6%	8	6	Egg whites, olive oil, salsa, cantaloupe, sweet potato, pineapple
**dish_1566316726**	30	17	13	56.7%	43.3%	8	3	Egg whites, olive oil, sweet potato
**dish_1566838378**	36	9	27	25.0%	75.0%	8	4	Scrambled eggs, watermelon, berries, pineapple
**dish_1566838407**	29	6	23	20.7%	79.3%	8	6	Pineapple, scrambled eggs, sweet potato, berries, olive oil, watermelon
***Chicken-based* group** (median number of ingredients per dish: 4, IQR: 3–5)								
**dish_1550706583**	114	87	27	76.3%	23.7%	7	1	Chicken
**dish_1550706705**	115	55	60	47.8%	52.2%	7	3	Chicken, cucumbers, cauliflower
**dish_1550873434**	119	119	0	100.0%	0.0%	7	1	Chicken
**dish_1550873668**	117	59	58	50.4%	49.6%	8	3	Avocado, chicken, cheese
**dish_1563389600**	35	17	18	48.6%	51.4%	8	2	Grilled chicken, cherry tomatoes
**dish_1565811061**	34	18	16	52.9%	47.1%	8	4	Chicken, carrot, spinach (raw), broccoli
**dish_1565811091**	92	55	37	59.8%	40.2%	7	5	Broccoli, spinach (raw), carrot, chicken, goat cheese
**dish_1565811139**	29	10	19	34.5%	65.5%	7	6	Carrot, goat cheese, olive oil, broccoli, spinach (raw), chicken
**dish_1565974409**	30	14	16	46.7%	53.3%	7	2	Chicken apple sausage, bacon
**dish_1566246513**	37	27	10	73.0%	27.0%	7	3	Feta cheese, mixed greens, chicken
**dish_1566246626**	37	26	11	70.3%	29.7%	8	6	Feta cheese, broccoli, mixed greens, chicken, olives, Caesar dressing

Dish ID	Total original frames	Left frames	Removed frames	Percentage of left frames	Percentage of removed frames	Number of algorithms wrongly predicting the dish	Number of ingredients per dish	List of ingredients per dish
**dish_1566328776**	35	11	24	31.4%	68.6%	7	3	Chicken, cherry tomatoes, pizza
**dish_1566328805**	31	11	20	35.5%	64.5%	8	4	Cherry tomatoes, pizza, chicken, pineapple
**dish_1566328831**	34	10	24	29.4%	70.6%	8	5	Pizza, cherry tomatoes, chicken, pineapple, olives
**dish_1566850031**	36	10	26	27.8%	72.2%	8	6	Olives, carrot, chicken, Caesar dressing, cherry tomatoes, onions
**dish_1568147009**	38	18	20	47.4%	52.6%	8	4	Broccoli, steak, mixed greens, chicken
**dish_1568147044**	36	18	18	50.0%	50.0%	8	5	Broccoli, steak, mixed greens, chicken, olive oil
***Western-inspired breakfast foods* group (median number of ingredients per dish: 2, IQR: 1–3)**								
**dish_1557937079**	819	127	692	15.5%	84.5%	8	1	Bacon
**dish_1558372948**	32	23	9	71.9%	28.1%	8	1	Almonds
**dish_1558373159**	32	4	28	12.5%	87.5%	8	3	Sausage, APPLES with peel, almonds
**dish_1558375886**	41	5	36	12.2%	87.8%	8	3	Almonds, sausage, grapes
**dish_1558544663**	33	17	16	51.5%	48.5%	8	1	Oatmeal
**dish_1558549806**	35	9	26	25.7%	74.3%	7	3	Almonds, white rice, spinach (raw)
**dish_1560203424**	28	11	17	39.3%	60.7%	8	2	Pepperoni pizza, brownies
**dish_1563305513**	35	15	20	42.9%	57.1%	7	1	Cheese pizza
**dish_1566316681**	87	15	72	17.2%	82.8%	8	2	Sweet potato, olive oil
***Corrected-portion-sizes* group (median number of ingredients per dish: 3, IQR: 2–3)**								
**dish_1551382179**	116	76	40	65.5%	34.5%	8	3	Asparagus, white rice, oatmeal
**dish_1551567508**	114	114	0	100.0%	0.0%	8	1	Olives
**dish_1551567573**	115	74	41	64.3%	35.7%	8	3	Olives, sausage, ham
All incorrectly predicted dishes								
**Minimum**	24.0	1.0	0.0	3.1%	0.0%	7.0	1.0	—
**Q1**	29.0	8.8	16.0	23.8%	49.9%	8.0	3.0	—
**Median**	32.0	12.5	23.0	39.0%	61.0%	8.0	6.0	—
**Q3**	37.0	18.3	27.0	50.1%	76.2%	8.0	14.5	—
**Maximum**	819.0	127.0	692.0	100.0%	96.9%	8.0	28.0	—

Dish ID	Total original frames	Left frames	Removed frames	Percentage of left frames	Percentage of removed frames	Number of algorithms wrongly predicting the dish	Number of ingredients per dish	List of ingredients per dish
**Mean**	61.2	21.9	39.3	38.9%	61.1%	7.8	8.9	—
**SD**	104.6	27.5	89.7	22.3%	22.3%	0.4	7.8	—

Table S6. Performance metrics for single target variables, algorithms, and available datasets, as well as median metrics across datasets, after frame filtering. Nutrition5k test set (n = 664).

Dataset and algorithm[1]	Mass (g)			Energy content (kcal)			Protein content (g)			Fat content (g)			Carbohydrates content (g)		
	RMSE	MAE	MAPE	RMSE	MAE	MAPE	RMSE	MAE	MAPE	RMSE	MAE	MAPE	RMSE	MAE	MAPE
**US FCDB—no correction**															
IncV3_2+1	312.07	44.79	23.81	507.75	76.28	30.58	11.37	6.61	42.06	47.25	6.86	50.96	29.70	9.17	48.88
IncV3_2+2	311.49	42.92	22.82	507.46	74.66	29.93	10.71	5.82	37.01	47.28	6.61	49.13	29.54	8.64	46.06
R101_2+1	**311.02**	**40.93**	**21.76**	**505.64**	**71.92**	**28.83**	**8.97**	**4.88**	**31.06**	**46.87**	**6.05**	**44.94**	29.03	7.27	38.74
R101_2+2	311.27	42.77	22.74	505.88	72.00	28.86	9.48	5.16	32.82	46.97	6.13	45.58	**29.02**	7.21	38.40
R50_2+1	311.07	42.06	22.36	506.08	73.08	29.29	9.44	5.29	33.65	46.93	6.27	46.62	**29.02**	7.07	37.70
R50_2+2	311.24	42.51	22.60	506.28	73.65	29.52	9.75	5.19	33.05	46.97	6.07	45.11	28.93	6.98	37.20
ViT-B-16_2+1	311.22	42.50	22.59	508.11	76.20	30.55	9.76	5.11	32.53	47.03	6.12	45.51	28.93	**6.59**	**35.13**
ViT-B-16_2+2	311.04	42.74	22.72	506.91	76.45	30.65	10.00	5.53	35.18	47.00	6.47	48.11	29.33	7.99	42.57
**US FCDB—correction**															
IncV3_2+1	48.61	31.88	18.11	78.07	49.77	22.54	10.91	6.47	41.96	7.37	4.76	43.76	11.64	7.67	44.98
IncV3_2+2	48.54	32.77	18.62	74.66	48.55	21.98	10.77	6.70	43.51	6.81	4.28	39.34	12.13	8.35	48.98
R101_2+1	41.48	27.51	15.63	68.26	42.39	19.19	**8.24**	4.75	30.83	6.06	3.69	33.93	8.22	5.17	30.31
R101_2+2	**41.39**	**27.47**	**15.60**	**67.67**	**42.13**	**19.08**	8.32	4.67	30.30	**5.67**	3.44	31.62	8.53	5.24	30.70
R50_2+1	42.13	27.84	15.82	71.43	43.41	19.65	8.60	4.94	32.04	5.72	3.49	32.09	8.61	5.44	31.91
R50_2+2	42.35	28.01	15.91	68.76	43.36	19.63	8.32	4.68	30.36	5.61	3.41	31.34	8.52	5.28	30.97
ViT-B-16_2+1	45.46	30.52	17.33	78.72	47.47	21.49	8.49	4.81	31.20	5.99	3.63	33.32	7.85	4.81	28.21
ViT-B-16_2+2	43.95	29.55	16.79	77.97	47.71	21.60	7.99	**4.43**	**28.72**	**5.67**	**3.36**	**30.92**	**7.67**	**4.60**	**26.99**
**IT FCDB—no correction**															
IncV3_2+1	456.00	61.38	29.91	664.55	92.51	35.32	10.73	6.09	39.99	65.82	8.40	54.85	13.39	7.59	51.87
IncV3_2+2	455.66	61.48	29.96	664.56	92.13	35.18	11.81	6.93	45.56	66.05	8.31	54.25	14.92	8.94	61.11
R101_2+1	455.01	58.02	28.28	663.01	86.38	32.99	9.30	4.82	31.67	65.65	7.81	50.96	11.25	5.32	36.35
R101_2+2	**454.46**	**57.55**	**28.05**	**662.40**	88.11	33.65	9.49	5.05	33.21	65.67	7.54	49.23	11.92	5.99	40.98
R50_2+1	455.37	59.15	28.83	663.44	**86.67**	**33.10**	9.63	4.77	31.31	65.71	7.38	48.20	11.52	5.79	39.59
R50_2+2	455.32	59.24	28.87	662.48	87.05	33.24	9.46	4.78	31.44	**65.57**	7.46	48.72	11.34	5.61	38.37
ViT-B-16_2+1	454.54	58.00	28.27	663.35	87.31	33.34	**9.21**	4.67	30.71	65.65	7.27	47.48	10.15	4.74	32.39
ViT-B-16_2+2	455.02	58.43	28.48	663.38	87.19	33.29	9.24	**4.45**	**29.22**	65.63	**7.14**	**46.61**	**10.02**	**4.62**	**31.59**

Dataset and algorithm[1]	Mass (g)			Energy content (kcal)			Protein content (g)			Fat content (g)			Carbohydrates content (g)		
	RMSE	MAE	MAPE	RMSE	MAE	MAPE	RMSE	MAE	MAPE	RMSE	MAE	MAPE	RMSE	MAE	MAPE
**IT FCDB—correction**															
IncV3_2+1	47.04	31.34	17.79	91.16	52.78	23.56	9.92	5.89	39.71	8.65	4.76	40.63	12.77	7.56	53.17
IncV3_2+2	48.92	31.95	18.13	88.91	52.62	23.49	10.63	6.51	43.83	8.35	4.74	40.45	13.54	8.46	59.50
R101_2+1	43.01	28.72	16.30	83.17	48.09	21.47	7.27	4.61	31.03	7.10	3.76	32.11	9.33	5.20	36.54
R101_2+2	**41.56**	**27.13**	**15.40**	86.80	**47.30**	**21.12**	9.05	5.12	34.46	8.13	4.17	35.60	11.44	6.81	47.87
R50_2+1	41.92	27.58	15.65	83.69	48.52	21.66	7.81	4.70	31.67	7.23	3.88	33.10	10.37	5.45	38.33
R50_2+2	43.44	28.54	16.19	84.65	49.37	22.04	7.41	4.34	29.24	7.16	3.81	32.56	9.74	5.00	35.19
ViT-B-16_2+1	44.52	29.46	16.72	**82.59**	48.50	21.65	7.47	4.46	30.04	**6.53**	3.85	32.92	**8.37**	4.35	30.58
ViT-B-16_2+2	45.60	30.28	17.18	86.35	50.44	22.52	**6.88**	**4.03**	**27.13**	6.85	**3.63**	**31.04**	**8.37**	**4.19**	**29.43**
**Overall median**															
IncV3_2+1	180.34	38.33	20.96	299.45	64.53	27.07	10.82	6.28	40.98	27.95	5.81	47.36	13.08	7.63	50.37
IncV3_2+2	180.20	37.85	20.72	298.19	63.64	26.71	10.74	6.61	43.67	27.82	5.67	44.79	14.23	8.55	54.24
R101_2+1	177.01	**34.82**	**19.03**	**294.40**	60.01	25.15	**8.61**	4.79	31.05	26.99	**4.90**	39.43	10.29	5.26	36.45
R101_2+2	**176.41**	35.12	19.17	296.34	**59.65**	**24.99**	9.26	5.08	33.01	27.55	5.15	40.59	11.68	6.40	39.69
R50_2+1	176.60	34.95	19.09	294.89	60.80	25.48	9.02	4.85	31.86	27.08	5.07	39.86	10.95	5.62	38.02
R50_2+2	177.34	35.52	19.40	295.47	61.51	25.78	8.89	4.73	30.90	27.07	4.94	38.83	10.54	5.45	36.19
ViT-B-16_2+1	178.34	36.51	19.96	295.35	62.35	26.10	8.85	4.74	30.95	**26.78**	4.99	39.41	9.26	4.78	31.49
ViT-B-16_2+2	178.32	36.51	19.95	296.63	63.45	26.58	8.62	**4.44**	**28.97**	26.93	5.05	**38.82**	**9.20**	**4.61**	**30.51**

[1]US FCDB—no correction was directly obtained by using the original file from the Nutrition5k project; US FCDB—correction was obtained from US FCDB—no correction by carrying out correction of portion sizes for selected ingredients in dishes; IT FCDB—no correction was obtained by substituting the nutritional composition from the US FCDB with that from the IT FCDB and this included imputing missing-name ingredients, trace values, and missing values for food items in the Italian FCDB; IT FCDB—correction was obtained after correcting portion sizes for selected ingredients in dishes. Values in bold typeface with a light blue cell background indicate the minimum performance metrics (i.e., the minimum error) for each target variable; similarly values with an orange cell background indicate the maximum performance MAPE (i.e., the maximum error) for each target variable as commented in the text. Abbreviations: FCDB, Food Composition Database

Table S7. Sensitivity analysis: percentages of perfect agreement of pairs of predicted and computed energy contents under the 4-task and 5-task deep learning algorithms, before and after frame filtering. Nutrition5k test set (n = 676 and n = 664, respectively).

	BEFORE FRAME FILTERING			AFTER FRAME FILTERING		
**Dataset and algorithm**[1]	**5-Task Computed vs. 4-Task Computed Energy Content**	**5-Task Predicted vs. 4-Task Computed Energy Content**	**5-Task Predicted vs. 5-Task Computed Energy Content**	**5-Task Computed vs. 4-Task Computed Energy Content**	**5-Task Predicted vs. 4-Task Computed Energy Content**	**5-Task Predicted vs. 5-Task Computed Energy Content**
**US FCDB—no correction**						
IncV3_2+1	**0.666**	0.648	**0.910**	0.643	0.628	0.895
IncV3_2+2	0.670	**0.586**	0.679	**0.590**	**0.581**	0.878
R101_2+1	0.663	0.703	0.821	0.643	0.687	0.789
R101_2+2	0.698	0.667	0.898	0.690	0.651	**0.896**
R50_2+1	0.716	0.741	0.858	0.697	0.721	**0.843**
R50_2+2	0.728	0.723	0.891	0.708	0.699	0.880
ViT-B-16_2+1	0.809	**0.775**	0.892	**0.803**	**0.764**	0.870
ViT-B-16_2+2	**0.827**	0.601	**0.593**	0.614	0.610	0.893
**US FCDB—correction**						
IncV3_2+1	0.660	0.660	**0.833**	0.652	0.652	0.837
IncV3_2+2	**0.614**	**0.604**	0.902	**0.608**	**0.593**	0.898
R101_2+1	0.706	0.660	0.855	0.693	0.633	**0.834**
R101_2+2	0.670	0.691	0.882	0.658	0.682	0.886
R50_2+1	0.722	0.698	0.849	0.714	0.690	**0.834**
R50_2+2	0.719	0.695	**0.905**	0.702	0.682	**0.901**
ViT-B-16_2+1	0.788	**0.780**	0.879	0.788	**0.780**	0.878
ViT-B-16_2+2	**0.812**	0.775	0.864	**0.813**	0.771	0.858
**IT FCDB—no correction**						
IncV3_2+1	0.661	0.667	**0.939**	0.669	0.670	**0.940**
IncV3_2+2	**0.643**	**0.638**	0.828	**0.640**	**0.625**	0.819
R101_2+1	0.706	0.744	0.885	0.690	0.721	0.870
R101_2+2	0.695	0.649	**0.814**	0.690	0.642	**0.813**
R50_2+1	0.700	0.709	0.842	0.682	0.697	**0.813**
R50_2+2	0.710	0.707	0.867	0.699	0.696	0.861
ViT-B-16_2+1	0.769	0.784	0.911	0.759	0.785	0.904
ViT-B-16_2+2	**0.812**	**0.802**	0.917	**0.810**	**0.794**	0.925

	BEFORE FRAME FILTERING			AFTER FRAME FILTERING		
**Dataset and algorithm**[1]	**5-Task Computed vs. 4-Task Computed Energy Content**	**5-Task Predicted vs. 4-Task Computed Energy Content**	**5-Task Predicted vs. 5-Task Computed Energy Content**	**5-Task Computed vs. 4-Task Computed Energy Content**	**5-Task Predicted vs. 4-Task Computed Energy Content**	**5-Task Predicted vs. 5-Task Computed Energy Content**
**IT FCDB—correction**						
IncV3_2+1	**0.484**	0.614	**0.714**	**0.474**	**0.610**	**0.697**
IncV3_2+2	0.611	**0.601**	0.852	0.623	**0.610**	0.846
R101_2+1	0.683	0.741	0.836	0.679	0.747	0.840
R101_2+2	0.652	0.632	0.814	0.631	0.623	0.810
R50_2+1	0.709	0.703	0.855	0.708	0.700	0.839
R50_2+2	0.710	0.706	0.867	0.712	0.696	0.870
ViT-B-16_2+1	**0.765**	**0.768**	**0.920**	**0.767**	**0.765**	0.904
ViT-B-16_2+2	0.738	0.737	0.917	0.736	0.745	**0.913**

[1]US FCDB—no correction was directly obtained by using the original file from the Nutrition5k project; US FCDB—correction was obtained from US FCDB—no correction by carrying out correction of portion sizes for selected ingredients in dishes; IT FCDB—no correction was obtained by substituting the nutritional composition from the US FCDB with that from the IT FCDB and this included imputing missing-name ingredients, trace values, and missing values for food items in the Italian FCDB; IT FCDB—correction was obtained after correcting portion sizes for selected ingredients in dishes. Values in bold typeface with a light blue cell background indicate the minimum and maximum performance metrics for each target variable. Abbreviations: FCDB, Food Composition Database

Table S8. Sensitivity analysis: unweighted Cohen's kappa coefficients of pairs of predicted and computed energy contents under the 4-task and 5-task deep learning algorithms, before and after frame filtering. Nutrition5k test set (*n* = 676 and *n* = 664, respectively).

	BEFORE FRAME FILTERING			AFTER FRAME FILTERING		
**Dataset and algorithm[1]**	**5-Task Computed vs. 4-Task Computed Energy Content**	**5-Task Predicted vs. 4-Task Computed Energy Content**	**5-Task Predicted vs. 5-Task Computed Energy Content**	**5-Task Computed vs. 4-Task Computed Energy Content**	**5-Task Predicted vs. 4-Task Computed Energy Content**	**5-Task Predicted vs. 5-Task Computed Energy Content**
**US FCDB—no correction**						
IncV3_2+1	0.554*	0.531*	**0.880***	0.524*	0.504*	0.859*
IncV3_2+2	0.560*	**0.448***	0.572*	**0.454***	**0.442***	0.837*
R101_2+1	**0.550***	0.604*	0.761*	0.524*	0.582*	**0.719***
R101_2+2	0.598*	0.556*	0.864*	0.586*	0.534*	**0.861***
R50_2+1	0.621*	0.655*	0.811*	0.596*	0.629*	0.791*
R50_2+2	0.637*	0.631*	0.854*	0.61*	0.598*	0.839*
ViT-B-16_2+1	0.746*	**0.700***	0.856*	**0.737***	**0.685***	0.827*
ViT-B-16_2+2	**0.769***	0.467*	**0.458***	0.486*	0.480*	0.857*
**US FCDB—correction**						
IncV3_2+1	0.546*	0.546*	**0.777***	0.536*	0.536*	0.783*
IncV3_2+2	**0.485***	**0.471***	0.870*	**0.478***	**0.458***	0.863*
R101_2+1	0.607*	0.546*	0.807*	0.590*	0.510*	**0.779***
R101_2+2	0.560*	0.588*	0.842*	0.544*	0.576*	0.847*
R50_2+1	0.629*	0.598*	0.799*	0.618*	0.586*	**0.779***
R50_2+2	0.625*	0.594*	**0.874***	0.602*	0.576*	**0.867***
ViT-B-16_2+1	0.718*	**0.706***	0.838*	0.717*	**0.707***	0.837*
ViT-B-16_2+2	**0.750***	0.700*	0.819*	**0.751***	0.695*	0.811*
**IT FCDB—no correction**						
IncV3_2+1	0.548*	0.556*	**0.919***	0.558*	0.560*	**0.920***
IncV3_2+2	**0.525***	**0.517***	0.771*	**0.520***	**0.500***	0.759*
R101_2+1	0.607*	0.659*	0.846*	0.586*	0.629*	0.827*
R101_2+2	0.594*	0.533*	**0.751***	0.586*	0.522*	**0.751***
R50_2+1	0.600*	0.611*	0.789*	0.576*	0.596*	**0.751***
R50_2+2	0.613*	0.609*	0.822*	0.598*	0.594*	0.815*
ViT-B-16_2+1	0.692*	0.712*	0.882*	0.679*	0.713*	0.871*
ViT-B-16_2+2	**0.750***	**0.736***	0.89*	**0.747***	**0.725***	0.900*

	BEFORE FRAME FILTERING			AFTER FRAME FILTERING		
**Dataset and algorithm**[1]	**5-Task Computed vs. 4-Task Computed Energy Content**	**5-Task Predicted vs. 4-Task Computed Energy Content**	**5-Task Predicted vs. 5-Task Computed Energy Content**	**5-Task Computed vs. 4-Task Computed Energy Content**	**5-Task Predicted vs. 4-Task Computed Energy Content**	**5-Task Predicted vs. 5-Task Computed Energy Content**
**IT FCDB—correction**						
IncV3_2+1	**0.312***	0.485*	**0.619***	**0.299***	**0.480***	**0.596***
IncV3_2+2	0.481*	**0.467***	0.803*	0.498*	**0.480***	0.795*
R101_2+1	0.578*	0.655*	0.781*	0.572*	0.663*	0.787*
R101_2+2	0.536*	0.509*	0.751*	0.508*	0.498*	0.747*
R50_2+1	0.611*	0.604*	0.807*	0.610*	0.600*	0.785*
R50_2+2	0.613*	0.607*	0.822*	0.616*	0.594*	0.827*
ViT-B-16_2+1	**0.686***	**0.690***	**0.893***	**0.689***	**0.687***	0.871*
ViT-B-16_2+2	0.651*	0.649*	0.890*	0.649*	0.661*	**0.884***

[1]US FCDB—no correction was directly obtained by using the original file from the Nutrition5k project; US FCDB—correction was obtained from US FCDB—no correction by carrying out correction of portion sizes for selected ingredients in dishes; IT FCDB—no correction was obtained by substituting the nutritional composition from the US FCDB with that from the IT FCDB and this included imputing missing-name ingredients, trace values, and missing values for food items in the Italian FCDB; IT FCDB—correction was obtained after correcting portion sizes for selected ingredients in dishes. Values in bold typeface with a light blue cell background indicate the minimum and maximum performance metrics for each target variable. Abbreviations: FCDB, Food Composition Database

Table S9. Sensitivity analysis: performance metrics for predicted and computed energy contents under the 4-task and 5-task deep learning algorithms, before and after frame filtering. Nutrition5k test set (*n* = 676 and *n* = 664, respectively).

BEFORE FRAME FILTERING									
	5-Task Predicted Energy Content			5-Task Computed Energy Content			4-Task Computed Energy Content		
**Target variable and algorithm[1]**	**RMSE**	**MAE**	**MAPE**	**RMSE**	**MAE**	**MAPE**	**RMSE**	**MAE**	**MAPE**
**Mass (g)**									
IncV3_2+1	183.41	41.50	22.57	183.41	41.50	22.57	183.62	41.83	22.75
IncV3_2+2	182.73	40.11	21.84	183.33	41.80	22.74	184.39	41.93	22.80
R101_2+1	180.22	**37.69**	**20.49**	180.22	**37.69**	**20.49**	180.13	**37.25**	**20.24**
R101_2+2	**179.19**	37.74	**20.49**	**179.19**	37.74	**20.49**	180.50	37.60	20.45
R50_2+1	179.75	38.00	20.64	179.75	38.00	20.64	180.69	38.87	21.12
R50_2+2	180.42	38.48	20.90	180.42	38.48	20.90	180.25	38.87	21.12
ViT-B-16_2+1	180.12	38.53	20.95	180.12	38.53	20.95	180.46	38.49	20.92
ViT-B-16_2+2	180.50	38.58	20.97	180.53	38.77	21.07	**179.78**	38.42	20.89
**Energy content (kcal)**									
IncV3_2+1	302.71	69.19	28.68	311.86	76.27	31.75	312.49	83.51	34.71
IncV3_2+2	301.16	67.90	28.17	305.45	70.92	29.44	320.05	89.55	37.28
R101_2+1	**296.36**	**63.66**	**26.36**	295.58	65.49	27.27	299.84	68.94	28.54
R101_2+2	298.33	63.75	26.39	302.29	67.08	27.85	304.97	72.73	30.22
R50_2+1	297.25	64.70	26.79	300.51	68.42	28.34	300.98	70.01	28.99
R50_2+2	297.43	65.13	26.97	**298.15**	**65.37**	**27.07**	302.14	70.90	29.38
ViT-B-16_2+1	297.73	65.50	27.12	298.99	69.63	28.95	297.70	67.85	28.08
ViT-B-16_2+2	299.41	66.49	27.55	301.23	67.69	28.05	**297.23**	**67.16**	**27.78**
**Protein content (g)**									
IncV3_2+1	11.52	6.71	43.77	11.52	6.71	43.77	10.85	6.61	43.32
IncV3_2+2	11.41	7.05	46.61	11.90	7.17	46.61	11.24	6.97	45.64
R101_2+1	9.40	5.21	33.76	9.40	5.21	33.76	9.72	5.32	34.70
R101_2+2	9.90	5.46	35.62	9.90	5.46	35.62	10.19	5.73	37.06
R50_2+1	9.83	5.30	34.74	9.83	5.30	34.74	9.61	5.43	35.44
R50_2+2	9.71	5.17	33.77	9.71	5.17	33.77	9.71	5.36	35.00

ViT-B-16_2+1	9.49	5.10	33.27	9.49	5.10	33.27	9.02	4.76	31.10
ViT-B-16_2+2	**9.29**	**4.80**	**31.32**	**9.29**	**4.80**	**31.32**	**8.92**	**4.62**	**30.14**
**Fat content (g)**									
IncV3_2+1	27.95	6.03	48.56	27.95	6.03	48.56	28.55	6.88	53.73
IncV3_2+2	27.72	5.81	45.33	27.91	5.97	46.47	29.39	7.45	58.34
R101_2+1	26.93	**5.10**	40.80	26.93	5.10	40.80	27.13	5.28	41.44
R101_2+2	27.47	5.35	41.63	27.47	5.35	41.63	28.03	5.86	45.87
R50_2+1	27.10	5.29	41.02	27.10	5.29	41.02	27.29	5.56	43.10
R50_2+2	27.04	5.12	**39.75**	27.04	5.12	39.75	27.39	5.54	43.05
ViT-B-16_2+1	**26.82**	5.16	40.52	**26.82**	5.16	40.52	**26.68**	5.06	40.16
ViT-B-16_2+2	27.02	5.27	39.97	26.99	**4.99**	**38.89**	**26.68**	**4.96**	**38.89**
**Carbohydrates content (g)**									
IncV3_2+1	13.80	8.00	51.23	13.80	8.00	51.23	14.05	8.29	52.34
IncV3_2+2	14.89	8.72	54.76	14.89	9.04	56.58	15.16	9.25	57.54
R101_2+1	11.00	5.63	37.96	11.00	5.63	37.96	11.12	5.97	38.39
R101_2+2	12.27	6.76	40.55	12.27	6.76	40.55	12.81	7.37	44.31
R50_2+1	11.67	6.01	39.13	11.67	6.01	39.13	11.85	5.99	39.67
R50_2+2	11.28	5.79	37.55	11.28	5.79	37.55	11.17	5.91	38.32
ViT-B-16_2+1	10.29	5.19	33.54	10.29	5.19	33.54	10.34	5.09	32.71
ViT-B-16_2+2	**10.20**	**5.01**	**32.62**	**10.20**	**5.01**	**32.62**	**10.16**	**4.96**	**31.76**
**Overall median**									
IncV3_2+1	27.95	8.00	43.77	27.95	8.00	43.77	28.55	8.29	43.32
IncV3_2+2	27.72	8.72	45.33	27.91	9.04	46.47	29.39	9.25	45.64
R101_2+1	26.93	5.63	33.76	26.93	5.63	33.76	27.13	5.97	34.70
R101_2+2	27.47	6.76	35.62	27.47	6.76	35.62	28.03	7.37	37.06
R50_2+1	27.10	6.01	34.74	27.10	6.01	34.74	27.29	5.99	35.44
R50_2+2	27.04	5.79	33.77	27.04	5.79	33.77	27.39	5.91	35.00
ViT-B-16_2+1	**26.82**	**5.19**	33.27	**26.82**	5.19	33.27	**26.68**	5.09	31.10
ViT-B-16_2+2	27.02	5.27	**31.32**	26.99	**5.01**	**31.32**	**26.68**	**4.96**	**30.14**

**AFTER FRAME FILTERING**									
	**5-Task Predicted**			**5-Task Computed**			**4-Task Computed**		

Target variable and algorithm[1]	Energy Content			Energy Content			Energy Content		
	RMSE	MAE	MAPE	RMSE	MAE	MAPE	RMSE	MAE	MAPE
**Mass (g)**									
IncV3_2+1	180.34	38.33	20.96	180.34	38.33	20.96	180.89	38.76	21.19
IncV3_2+2	180.20	37.85	20.72	180.20	37.85	20.72	181.56	38.87	21.25
R101_2+1	177.01	**34.82**	**19.03**	177.01	**34.82**	**19.03**	**177.57**	**34.33**	**18.76**
R101_2+2	**176.41**	35.12	19.17	**176.41**	35.12	19.17	178.19	34.86	19.07
R50_2+1	176.60	34.95	19.09	176.60	34.95	19.09	178.23	36.03	19.69
R50_2+2	177.34	35.52	19.40	177.34	35.52	19.40	177.87	36.16	19.76
ViT-B-16_2+1	178.34	36.51	19.96	178.34	36.51	19.96	178.17	36.04	19.70
ViT-B-16_2+2	178.32	36.51	19.95	178.32	36.51	19.95	177.76	36.10	19.74
**Energy content (kcal)**									
IncV3_2+1	299.45	64.53	27.07	309.15	71.75	30.24	309.69	79.25	33.35
IncV3_2+2	298.19	63.64	26.71	301.20	65.70	27.60	316.92	84.99	35.83
R101_2+1	**294.40**	60.01	25.15	**293.81**	62.15	26.05	297.87	65.39	27.39
R101_2+2	296.34	**59.65**	**24.99**	300.25	62.91	26.43	303.09	69.08	29.05
R50_2+1	294.89	60.80	25.48	298.12	64.45	27.01	299.20	66.40	27.82
R50_2+2	295.47	61.51	25.78	295.87	**61.58**	**25.80**	300.69	67.50	28.30
ViT-B-16_2+1	295.35	62.35	26.10	296.36	66.19	27.89	294.92	64.13	26.85
ViT-B-16_2+2	296.63	63.45	26.58	298.23	64.01	26.83	**294.25**	**63.46**	**26.70**
**Protein content (g)**									
IncV3_2+1	10.82	6.28	40.98	10.82	6.28	40.98	10.07	6.14	40.37
IncV3_2+2	10.74	6.61	43.67	10.74	6.61	43.67	10.46	6.50	42.55
R101_2+1	**8.61**	4.79	31.05	**8.61**	4.79	31.05	8.98	4.90	32.01
R101_2+2	9.26	5.08	33.01	9.26	5.08	33.01	9.40	5.28	34.19
R50_2+1	9.02	4.85	31.86	9.02	4.85	31.86	8.82	5.02	32.80
R50_2+2	8.89	4.73	30.90	8.89	4.73	30.90	8.92	4.92	32.12
ViT-B-16_2+1	8.85	4.74	30.95	8.85	4.74	30.95	8.30	4.37	28.51
ViT-B-16_2+2	8.62	**4.44**	**28.97**	8.62	**4.44**	**28.97**	**8.21**	**4.24**	**27.69**
**Fat content (g)**									
IncV3_2+1	27.95	5.81	47.36	27.95	5.81	47.36	28.51	6.68	52.84

IncV3_2+2	27.82	5.67	44.79	27.82	5.67	44.79	29.32	7.22	57.30
R101_2+1	26.99	**4.90**	39.43	26.99	**4.90**	39.43	27.14	5.08	40.18
R101_2+2	27.55	5.15	40.59	27.55	5.15	40.59	28.09	5.68	45.02
R50_2+1	27.08	5.07	39.86	27.08	5.07	39.86	27.33	5.36	42.09
R50_2+2	27.07	4.94	38.83	27.07	4.94	38.83	27.43	5.35	42.08
ViT-B-16_2+1	**26.78**	4.99	39.41	**26.78**	4.99	39.41	26.62	4.85	38.75
ViT-B-16_2+2	26.93	5.05	**38.82**	26.93	5.05	**38.82**	**26.61**	**4.73**	**37.25**
**Carbohydrates content (g)**									
IncV3_2+1	13.08	7.63	50.37	13.08	7.63	50.37	13.40	7.92	51.43
IncV3_2+2	14.23	8.55	54.24	14.23	8.55	54.24	14.58	8.94	57.06
R101_2+1	10.29	5.26	36.45	10.29	5.26	36.45	10.44	5.64	37.31
R101_2+2	11.68	6.40	39.69	11.68	6.40	39.69	12.19	7.05	43.60
R50_2+1	10.95	5.62	38.02	10.95	5.62	38.02	11.14	5.65	38.65
R50_2+2	10.54	5.45	36.19	10.54	5.45	36.19	10.44	5.57	37.27
ViT-B-16_2+1	9.26	4.78	31.49	9.26	4.78	31.49	9.34	4.67	30.77
ViT-B-16_2+2	**9.20**	**4.61**	**30.51**	**9.20**	**4.61**	**30.51**	**9.15**	**4.57**	**29.76**
**Overall median**									
IncV3_2+1	27.95	7.63	40.98	27.95	7.63	40.98	28.51	7.92	40.37
IncV3_2+2	27.82	8.55	43.67	27.82	8.55	43.67	29.32	8.94	42.55
R101_2+1	26.99	5.26	31.05	26.99	5.26	31.05	27.14	5.64	32.01
R101_2+2	27.55	6.40	33.01	27.55	6.40	33.01	28.09	7.05	34.19
R50_2+1	27.08	5.62	31.86	27.08	5.62	31.86	27.33	5.65	32.80
R50_2+2	27.07	5.45	30.90	27.07	5.45	30.90	27.43	5.57	32.12
ViT-B-16_2+1	**26.78**	**4.99**	30.95	**26.78**	**4.99**	30.95	26.62	4.85	28.51
ViT-B-16_2+2	26.93	5.05	**28.97**	26.93	5.05	**28.97**	**26.61**	**4.73**	**27.69**

[1]Values in bold typeface with a light blue cell background indicate the minimum performance metrics (i.e., the minimum error) for each target variable. Abbreviations: FCDB, Food Composition Database

Figure S1. Examples of food images, observed and predicted mass and nutritional composition for the different combinations of algorithms and available datasets.

Salad-based group

dish_1560800988

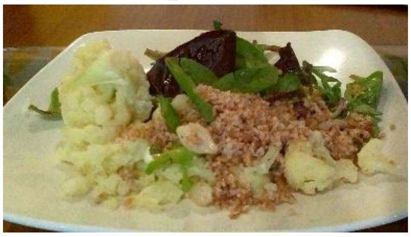

	Target Variable	Obseved	Predicted values							
		values	*IncV3_2+1*	*IncV3_2+2*	*R101_2+1*	*R101_2+2*	*R50_2+1*	*R50_2+2*	*ViT-B-16_2+1*	*ViT-B-16_2+2*
**US FCDB-no correction**	*Mass (g)*	230.00	254.34	282.10	296.78	293.88	278.58	284.74	233.33	282.10
	Energy (kcal)	314.87	269.64	338.03	336.49	324.79	334.26	333.08	211.46	338.03
	Protein (g)	11.73	18.70	24.24	24.28	24.14	24.88	23.57	12.56	24.24
	Fat (g)	5.12	13.79	17.92	17.71	15.96	17.76	16.47	11.76	17.92
	Carbohydrates (g)	61.25	19.60	22.07	25.97	23.46	22.59	25.60	16.41	22.07
**US FCDB-correction**	*Mass (g)*	230.00	258.65	273.19	272.21	278.91	267.49	274.02	229.97	222.88
	Energy (kcal)	314.87	260.21	295.56	295.46	295.99	296.19	303.07	195.79	195.40
	Protein (g)	11.73	17.04	22.25	20.35	21.27	21.90	22.27	11.22	12.15
	Fat (g)	5.12	12.27	13.63	13.26	14.79	14.70	14.74	10.32	10.68
	Carbohydrates (g)	61.25	16.78	20.32	23.16	21.47	23.15	22.15	17.04	16.60
**IT FCDB-no correction**	*Mass (g)*	230.00	264.75	273.86	280.58	278.89	268.77	283.66	235.65	227.80
	Energy (kcal)	141.74	299.30	294.66	303.60	317.44	296.76	330.57	222.26	220.50
	Protein (g)	7.35	20.76	19.91	22.07	22.85	19.58	22.52	13.34	12.38
	Fat (g)	4.18	16.35	14.06	14.70	13.94	13.00	15.14	11.24	11.17
	Carbohydrates (g)	15.32	15.17	16.29	18.10	20.15	19.82	23.03	15.61	15.97
**IT FCDB-correction**	*Mass (g)*	230.00	231.01	236.78	275.69	273.51	261.29	267.57	225.76	226.53
	Energy (kcal)	141.74	233.66	255.36	302.32	276.48	298.49	309.90	208.35	215.49
	Protein (g)	7.35	13.56	16.43	23.85	19.27	20.79	21.51	11.98	13.49
	Fat (g)	4.18	10.60	12.40	14.45	12.77	12.80	14.06	10.92	10.51
	Carbohydrates (g)	15.32	9.91	14.02	21.06	19.03	20.18	20.73	15.54	14.96

***Eggs-based* group**

dish_1566316726

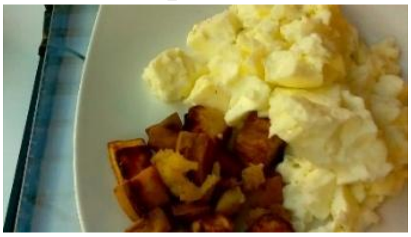

	Target Variable	Obseved	Predicted values							
		values	*IncV3_2+1*	*IncV3_2+2*	*R101_2+1*	*R101_2+2*	*R50_2+1*	*R50_2+2*	*ViT-B-16_2+1*	*ViT-B-16_2+2*
**US FCDB-no correction**	*Mass (g)*	685.00	450.84	567.74	566.43	553.44	562.59	563.74	527.79	567.74
	Energy (kcal)	409.52	350.34	389.07	386.34	382.87	371.69	379.00	342.15	389.07
	Protein (g)	57.49	33.28	40.91	48.49	43.28	44.06	49.37	42.14	40.91
	Fat (g)	4.10	11.46	10.37	8.22	9.03	8.21	7.76	5.31	10.37
	Carbohydrates (g)	25.37	27.33	28.54	27.37	28.31	27.94	25.78	19.18	28.54
**US FCDB-correction**	*Mass (g)*	685.00	498.32	490.20	556.82	537.37	539.19	534.89	522.98	538.44
	Energy (kcal)	409.52	349.19	357.59	371.43	380.49	348.97	360.39	341.55	342.95
	Protein (g)	57.49	31.46	31.95	43.27	45.81	46.48	44.01	41.75	46.19
	Fat (g)	4.10	9.24	9.58	6.12	9.70	6.21	7.34	4.52	4.70
	Carbohydrates (g)	25.37	26.85	30.93	27.33	24.24	24.73	25.25	22.76	20.74
**IT FCDB-no correction**	*Mass (g)*	685.00	487.25	466.91	555.74	575.91	513.10	545.14	556.57	516.75
	Energy (kcal)	378.34	283.91	304.15	335.74	377.29	317.96	337.64	325.48	302.26
	Protein (g)	61.67	28.92	21.74	48.21	49.57	42.01	45.39	48.18	45.49
	Fat (g)	3.61	7.32	8.03	7.37	8.25	5.59	6.68	5.07	5.04
	Carbohydrates (g)	25.63	26.32	28.84	17.58	23.30	20.71	23.05	22.29	19.13
**IT FCDB-correction**	*Mass (g)*	685.00	500.87	434.76	532.07	554.56	536.21	514.99	552.20	519.37
	Energy (kcal)	378.34	297.01	286.90	328.10	304.95	336.43	356.44	325.71	307.40
	Protein (g)	61.67	29.48	20.52	46.61	38.28	48.89	46.34	48.28	46.96
	Fat (g)	3.61	3.55	8.22	7.26	9.63	5.95	8.99	8.08	5.08
	Carbohydrates (g)	25.63	23.49	25.63	23.46	21.72	18.70	19.74	19.65	20.90

***Chicken-based* group**

dish_1565811061

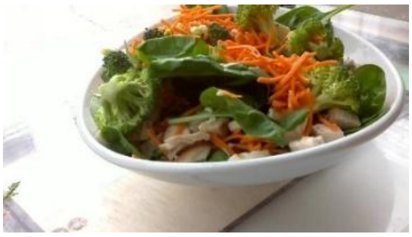

	Target Variable	Obseved	Predicted values							
		values	*IncV3_2+1*	*IncV3_2+2*	*R101_2+1*	*R101_2+2*	*R50_2+1*	*R50_2+2*	*ViT-B-16_2+1*	*ViT-B-16_2+2*
**US FCDB-no correction**	*Mass (g)*	324.00	254.34	282.10	296.78	293.88	278.58	284.74	233.33	282.10
	Energy (kcal)	380.32	269.64	338.03	336.49	324.79	334.26	333.08	211.46	338.03
	Protein (g)	65.80	18.70	24.24	24.28	24.14	24.88	23.57	12.56	24.24
	Fat (g)	7.76	13.79	17.92	17.71	15.96	17.76	16.47	11.76	17.92
	Carbohydrates (g)	9.06	19.60	22.07	25.97	23.46	22.59	25.60	16.41	22.07
**US FCDB-correction**	*Mass (g)*	324.00	258.65	273.19	272.21	278.91	267.49	274.02	229.97	222.88
	Energy (kcal)	380.32	260.21	295.56	295.46	295.99	296.19	303.07	195.79	195.40
	Protein (g)	65.80	17.04	22.25	20.35	21.27	21.90	22.27	11.22	12.15
	Fat (g)	7.76	12.27	13.63	13.26	14.79	14.70	14.74	10.32	10.68
	Carbohydrates (g)	9.06	16.78	20.32	23.16	21.47	23.15	22.15	17.04	16.60
**IT FCDB-no correction**	*Mass (g)*	324.00	264.75	273.86	280.58	278.89	268.77	283.66	235.65	227.80
	Energy (kcal)	370.49	299.30	294.66	303.60	317.44	296.76	330.57	222.26	220.50
	Protein (g)	59.95	20.76	19.91	22.07	22.85	19.58	22.52	13.34	12.38
	Fat (g)	11.42	16.35	14.06	14.70	13.94	13.00	15.14	11.24	11.17
	Carbohydrates (g)	5.78	15.17	16.29	18.10	20.15	19.82	23.03	15.61	15.97
**IT FCDB-correction**	*Mass (g)*	324.00	231.01	236.78	275.69	273.51	261.29	267.57	225.76	226.53
	Energy (kcal)	370.49	233.66	255.36	302.32	276.48	298.49	309.90	208.35	215.49
	Protein (g)	59.95	13.56	16.43	23.85	19.27	20.79	21.51	11.98	13.49
	Fat (g)	11.42	10.60	12.40	14.45	12.77	12.80	14.06	10.92	10.51
	Carbohydrates (g)	5.78	9.91	14.02	21.06	19.03	20.18	20.73	15.54	14.96

***Western-inspired breakfast foods* group**

dish_1558373159

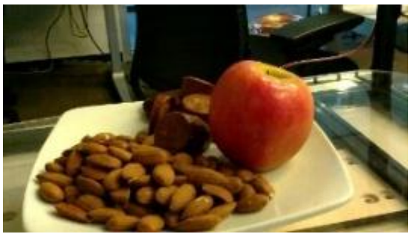

	Target Variable	Obseved	Predicted values							
		values	*IncV3_2+1*	*IncV3_2+2*	*R101_2+1*	*R101_2+2*	*R50_2+1*	*R50_2+2*	*ViT-B-16_2+1*	*ViT-B-16_2+2*
**US FCDB-no correction**	*Mass (g)*	334.00	299.99	294.60	292.87	304.22	279.94	309.69	270.63	294.60
	Energy (kcal)	1013.34	626.22	675.22	749.47	698.57	721.79	743.34	534.35	675.22
	Protein (g)	60.63	29.43	26.63	32.89	28.53	30.58	30.12	22.92	26.63
	Fat (g)	79.12	41.23	50.83	58.14	49.69	54.31	52.70	36.02	50.83
	Carbohydrates (g)	30.51	42.35	38.31	45.25	40.07	39.59	44.08	31.98	38.31
**US FCDB-correction**	*Mass (g)*	334.00	290.53	284.26	300.50	285.97	296.19	283.88	259.33	278.87
	Energy (kcal)	1013.34	626.63	671.40	717.57	721.89	709.27	725.37	511.85	512.49
	Protein (g)	60.63	25.47	37.30	28.50	29.00	31.44	30.25	22.25	22.88
	Fat (g)	79.12	38.74	39.91	50.31	53.17	49.83	52.09	35.97	35.18
	Carbohydrates (g)	30.51	34.26	34.85	39.10	38.52	41.36	39.47	32.98	34.29
**IT FCDB-no correction**	*Mass (g)*	334.00	289.92	309.59	301.47	313.34	304.50	298.77	267.05	259.39
	Energy (kcal)	1046.32	603.00	674.84	763.60	760.34	758.49	805.20	505.92	523.11
	Protein (g)	42.67	26.82	37.88	30.05	28.94	26.56	30.65	17.41	17.36
	Fat (g)	84.93	44.88	43.66	61.32	54.43	55.66	61.51	36.64	37.84
	Carbohydrates (g)	19.67	23.44	20.00	24.54	29.18	28.81	26.75	24.16	24.73
**IT FCDB-correction**	*Mass (g)*	334.00	282.24	286.94	301.95	296.81	290.04	273.07	262.90	258.00
	Energy (kcal)	1046.32	632.28	622.93	760.49	760.16	736.67	773.59	504.58	474.44
	Protein (g)	42.67	24.85	35.47	35.27	31.38	30.82	29.97	18.33	17.05
	Fat (g)	84.93	45.30	38.99	61.55	54.40	55.45	61.27	36.70	32.05
	Carbohydrates (g)	19.67	17.79	22.82	26.21	23.02	21.00	21.43	25.20	23.77

***Corrected-portion-sizes* group**

dish_1551567573

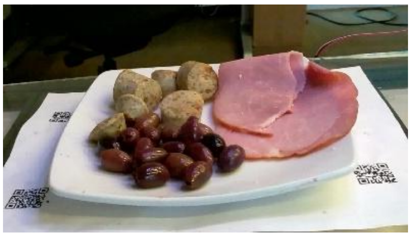

	Target Variable	Obseved	Predicted values							
		values	*IncV3_2+1*	*IncV3_2+2*	*R101_2+1*	*R101_2+2*	*R50_2+1*	*R50_2+2*	*ViT-B-16_2+1*	*ViT-B-16_2+2*
**US FCDB-no correction**	*Mass (g)*	159.00	150.59	167.58	156.74	168.94	161.92	162.53	168.36	167.58
	Energy (kcal)	9485.81	307.71	351.88	361.34	353.19	349.91	350.46	358.45	351.88
	Protein (g)	87.78	16.96	21.12	23.77	23.64	22.57	23.92	23.43	21.12
	Fat (g)	875.54	19.93	27.49	27.49	25.77	25.63	24.59	24.99	27.49
	Carbohydrates (g)	506.08	17.54	7.78	10.17	7.37	8.58	8.71	9.24	7.78
**US FCDB-correction**	*Mass (g)*	127.97	148.02	138.20	148.84	151.94	142.82	152.52	162.42	160.74
	Energy (kcal)	324.89	288.20	287.26	307.46	330.70	312.06	314.11	333.02	329.52
	Protein (g)	24.06	15.55	16.69	19.28	22.04	20.46	21.67	21.86	22.38
	Fat (g)	23.18	16.73	16.29	20.73	24.36	22.26	22.20	23.22	22.77
	Carbohydrates (g)	4.22	12.36	15.02	7.04	6.16	6.65	6.63	10.07	8.87
**IT FCDB-no correction**	*Mass (g)*	8094.00	155.48	166.86	178.20	187.29	174.71	179.42	181.62	177.45
	Energy (kcal)	12376.13	370.62	372.15	424.61	440.67	406.86	435.72	406.97	410.33
	Protein (g)	89.26	19.08	20.90	26.99	27.12	24.19	26.85	28.29	26.41
	Fat (g)	1221.83	29.72	24.01	34.54	33.28	30.94	35.72	30.66	31.25
	Carbohydrates (g)	80.18	5.34	10.69	1.77	3.38	2.96	1.56	5.68	3.89
**IT FCDB-correction**	*Mass (g)*	127.97	150.15	144.07	156.62	153.78	153.95	146.04	159.90	156.10
	Energy (kcal)	347.43	327.64	324.93	367.14	358.17	380.97	355.22	364.71	355.60
	Protein (g)	25.53	15.83	17.76	27.03	21.89	24.75	22.64	23.55	23.79
	Fat (g)	26.93	23.80	20.65	30.11	27.13	29.92	28.33	28.47	26.45
	Carbohydrates (g)	0.52	3.05	11.18	2.61	5.87	-0.26	0.76	4.56	2.67

Example of properly predicted dish

dish_1551224787

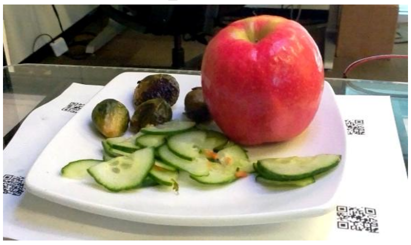

	Target Variable	Obseved	Predicted values							
		values	*IncV3_2+1*	*IncV3_2+2*	*R101_2+1*	*R101_2+2*	*R50_2+1*	*R50_2+2*	*ViT-B-16_2+1*	*ViT-B-16_2+2*
**US FCDB-no correction**	*Mass (g)*	330.00	235.40	265.81	262.59	272.87	265.13	270.57	257.82	265.81
	Energy (kcal)	151.15	124.79	142.84	137.96	143.70	137.41	144.86	144.93	142.84
	Protein (g)	2.43	2.44	2.91	3.18	2.66	3.23	3.04	3.76	2.91
	Fat (g)	0.61	1.66	1.81	2.01	1.25	2.22	1.90	2.34	1.81
	Carbohydrates (g)	39.45	28.38	33.24	30.62	33.25	29.89	32.35	27.89	33.24
**US FCDB-correction**	*Mass (g)*	330.00	235.52	220.57	264.90	265.97	290.68	262.74	251.23	270.55
	Energy (kcal)	151.15	123.74	117.34	133.71	136.61	148.93	143.12	128.79	147.61
	Protein (g)	2.43	0.95	3.46	2.49	2.83	3.65	3.08	3.35	3.64
	Fat (g)	0.61	1.41	0.72	0.95	1.03	1.45	1.13	1.45	1.62
	Carbohydrates (g)	39.45	27.21	22.90	31.34	30.16	34.70	31.48	26.31	30.36
**IT FCDB-no correction**	*Mass (g)*	331.00	245.07	239.15	282.48	274.67	287.72	280.42	263.26	253.62
	Energy (kcal)	167.70	132.92	149.12	144.84	149.76	149.35	144.50	153.15	148.18
	Protein (g)	2.60	3.73	7.61	3.42	4.48	1.90	2.99	5.11	3.47
	Fat (g)	0.65	2.14	3.20	2.19	1.03	0.89	1.01	3.04	2.26
	Carbohydrates (g)	36.87	23.68	16.31	24.58	23.95	28.51	27.10	24.67	26.06
**IT FCDB-correction**	*Mass (g)*	331.00	245.48	242.28	281.93	277.36	281.17	267.02	262.18	257.76
	Energy (kcal)	167.70	124.45	136.76	136.83	129.30	145.25	138.87	154.17	136.33
	Protein (g)	2.60	2.20	5.95	4.74	4.00	3.68	3.14	4.21	4.17
	Fat (g)	0.65	0.20	2.03	1.36	1.52	1.03	1.05	3.82	1.75
	Carbohydrates (g)	36.87	19.99	16.62	26.11	17.39	26.81	25.21	27.88	23.82

Figure S2. Representative training and validation loss curves supporting the choice of fixed training epochs. Rolling mean (window sizes of 3 and 5, respectively) was applied to smooth the curves for visualization purposes[1].

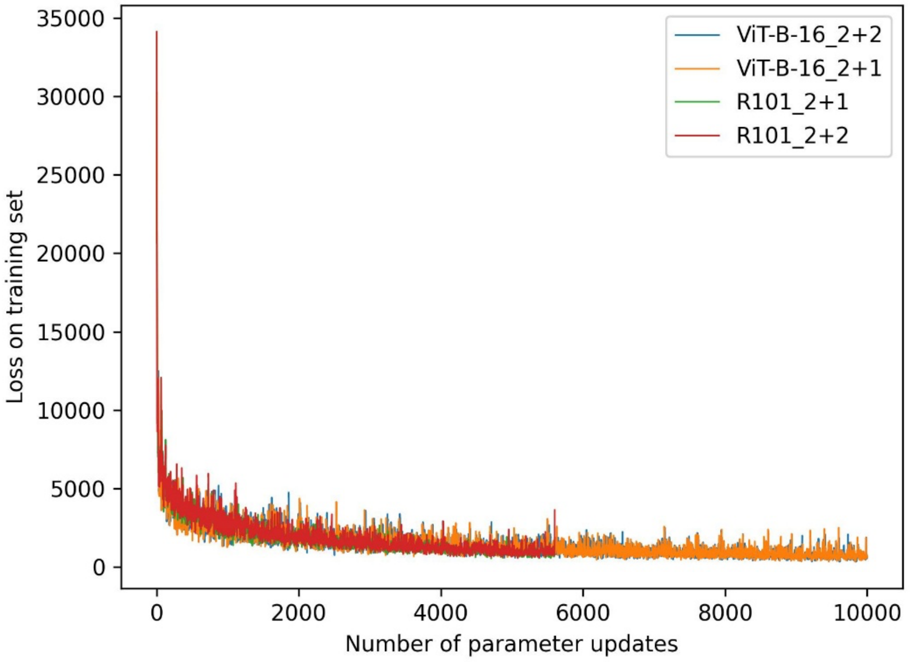

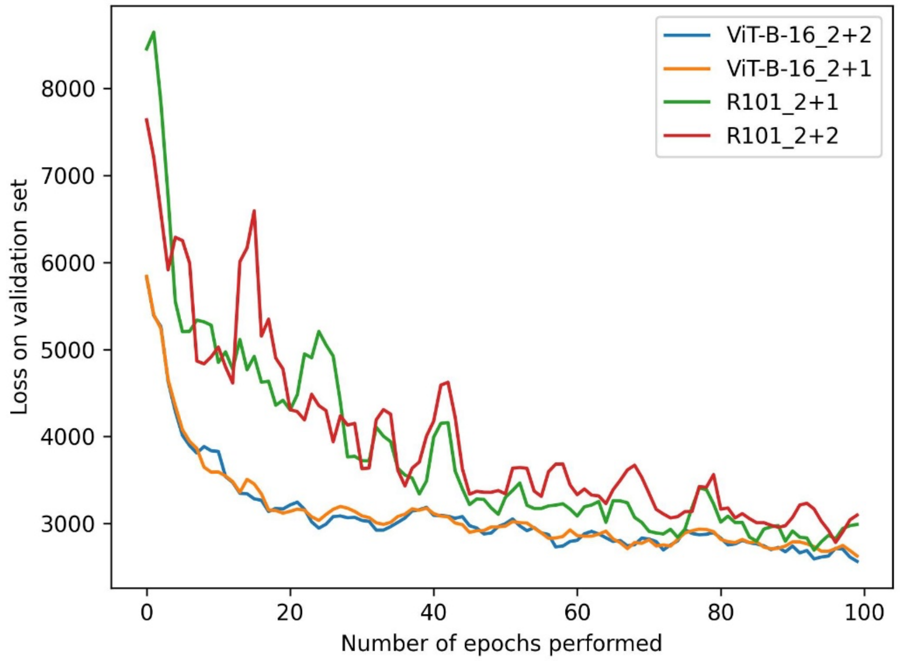

[1]ViT-B-16 was trained with a reduced batch size (half that of other models) due to higher memory requirements (see the Methods section for details). Consequently, the number of iterations per epoch is doubled, resulting in a greater number of loss values shown for this architecture in upper panel.

Figure S3-S7. Bland—Altman plots representing the raw absolute difference between predicted and observed values on the test set (y-axis) versus the mean of the predicted and observed values for each target variable (x-axis), with corresponding 95% limits of agreement. On the y-axis, the dotted line indicates the reference value of 0. Each panel represents mass, energy, or macronutrients content, and information is organized by algorithm and dataset in 32 plots. Nutrition5k test set (n = 676).

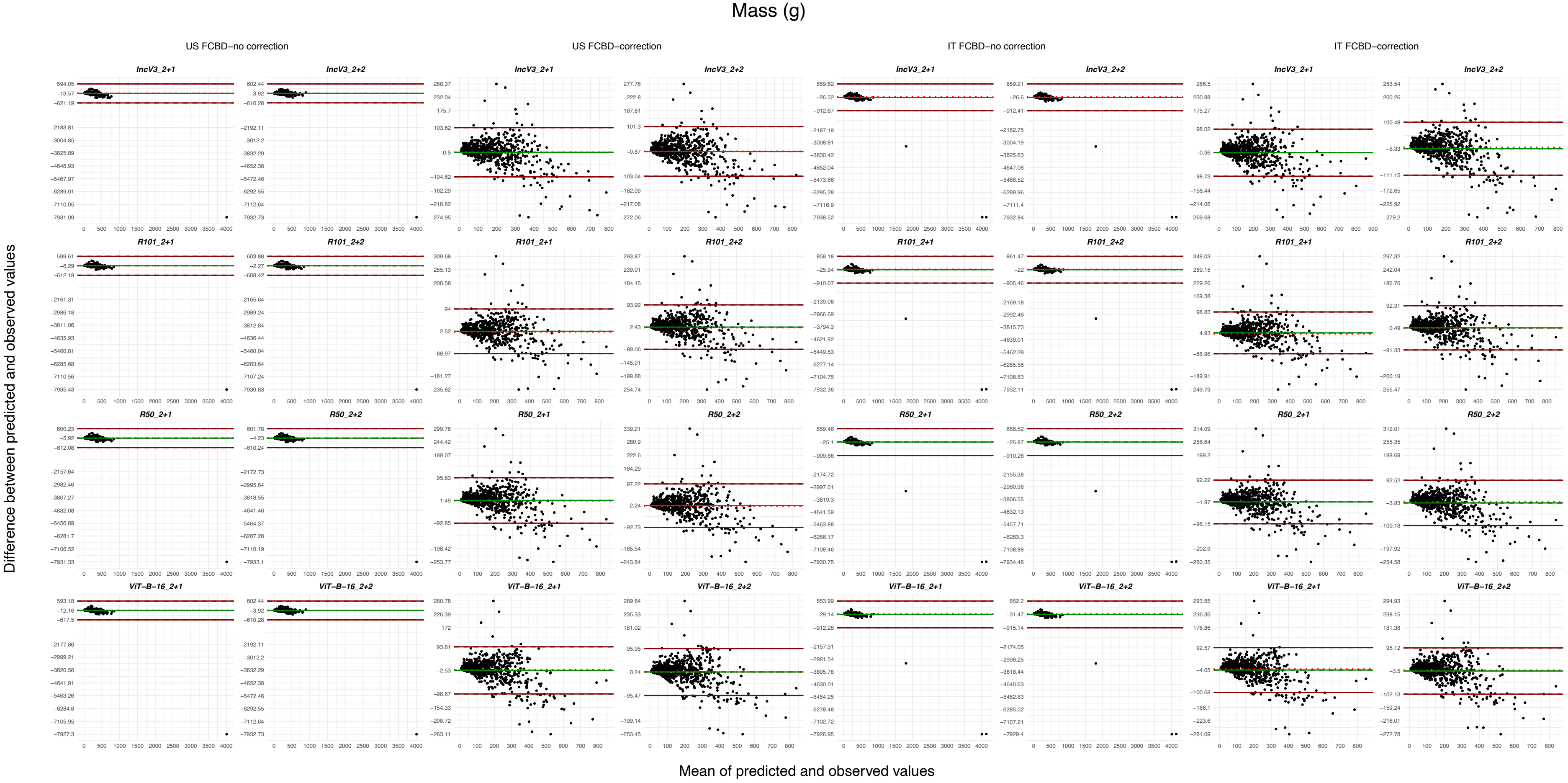

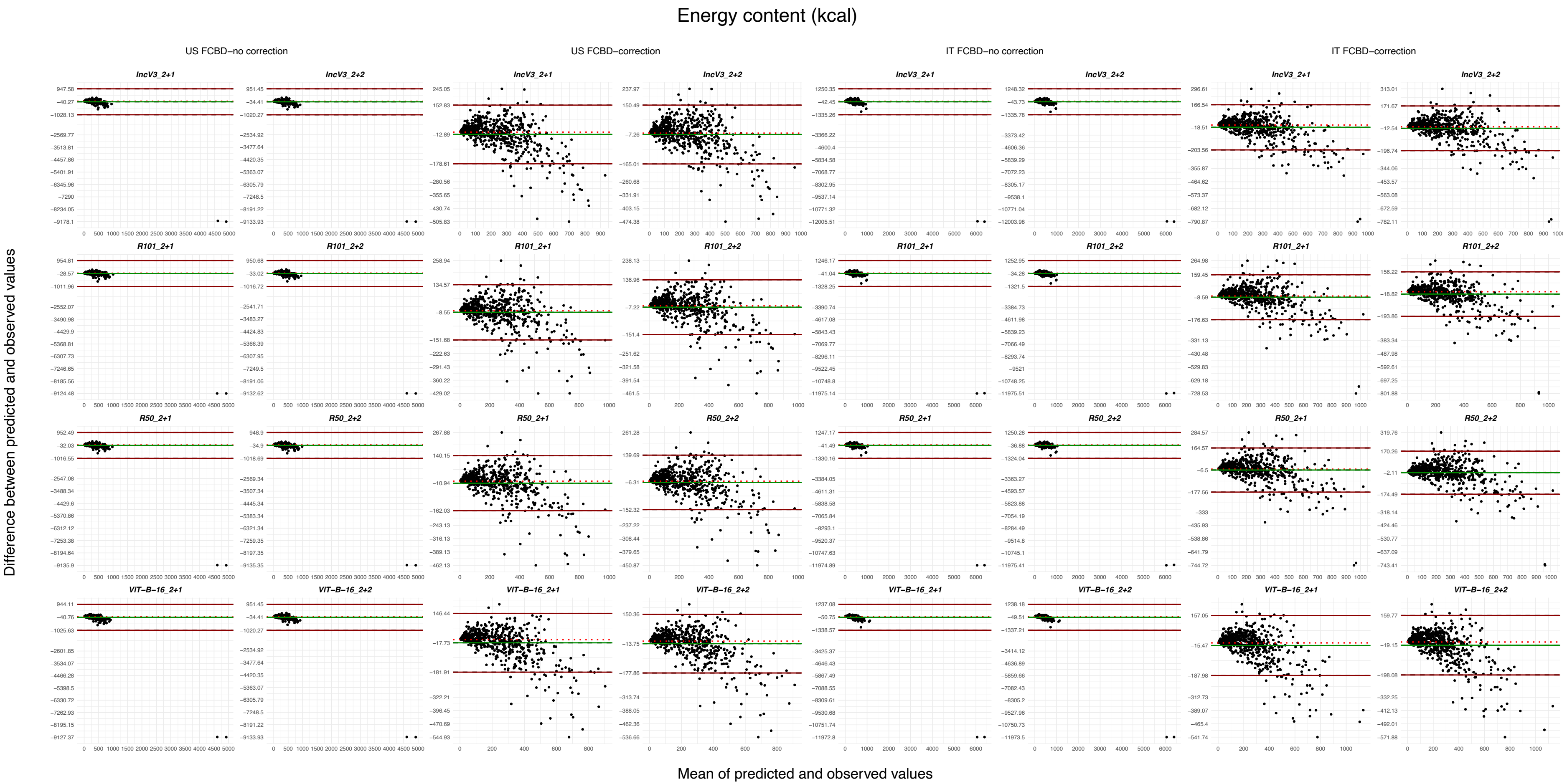
Energy content (kcal)
US FCBD–no correction
US FCBD–correction
IT FCBD–no correction
IT FCBD–correction
IncV3_2+1
IncV3_2+2
R101_2+1
R101_2+2
R50_2+1
R50_2+2
ViT–B–16_2+1
ViT–B–16_2+2
Difference between predicted and observed values
Mean of predicted and observed values

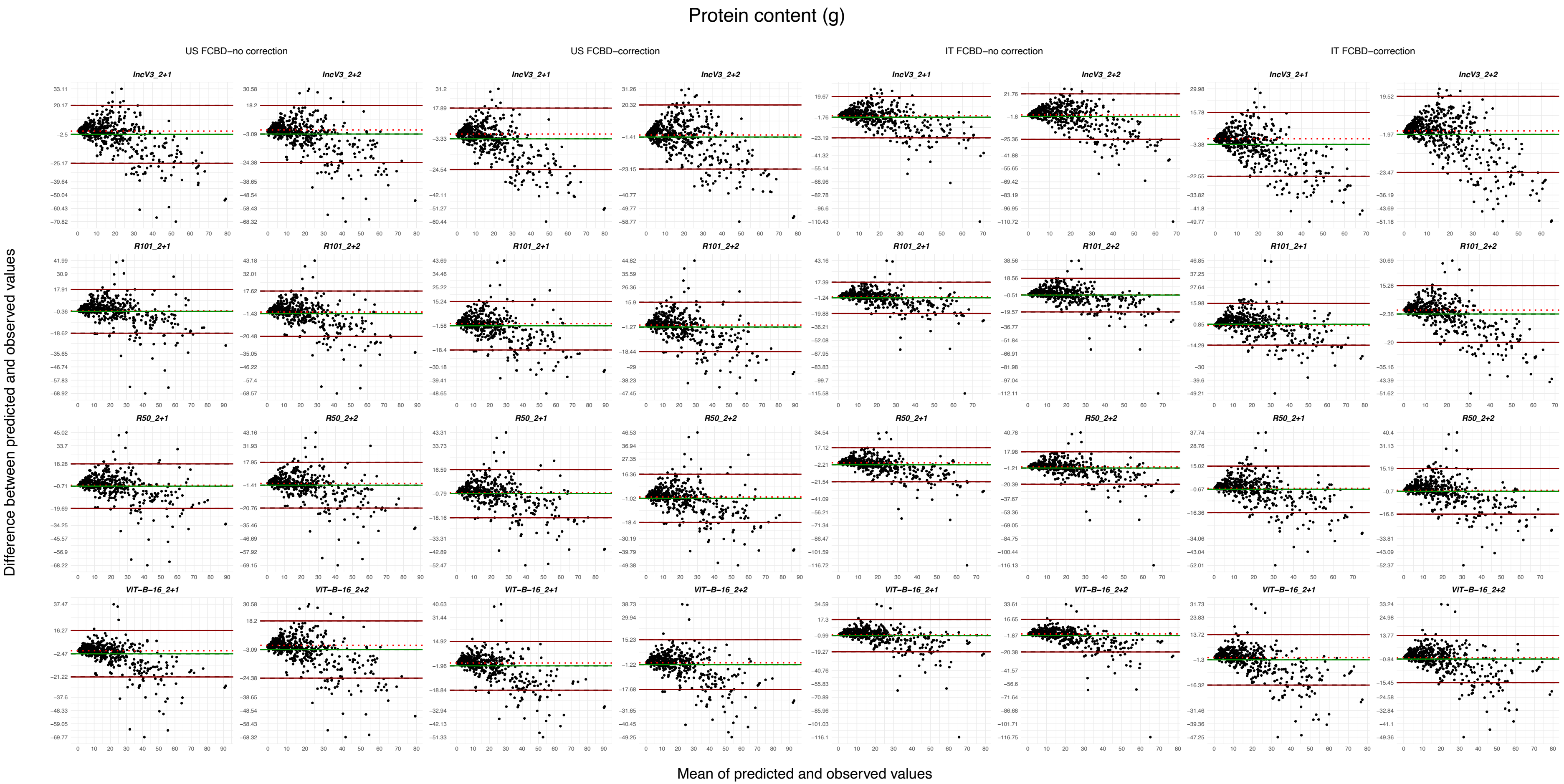
Protein content (g)
US FCBD–no correction
US FCBD–correction
IT FCBD–no correction
IT FCBD–correction
IncV3_2+1
IncV3_2+2
R101_2+1
R101_2+2
R50_2+1
R50_2+2
ViT–B–16_2+1
ViT–B–16_2+2
Difference between predicted and observed values
Mean of predicted and observed values

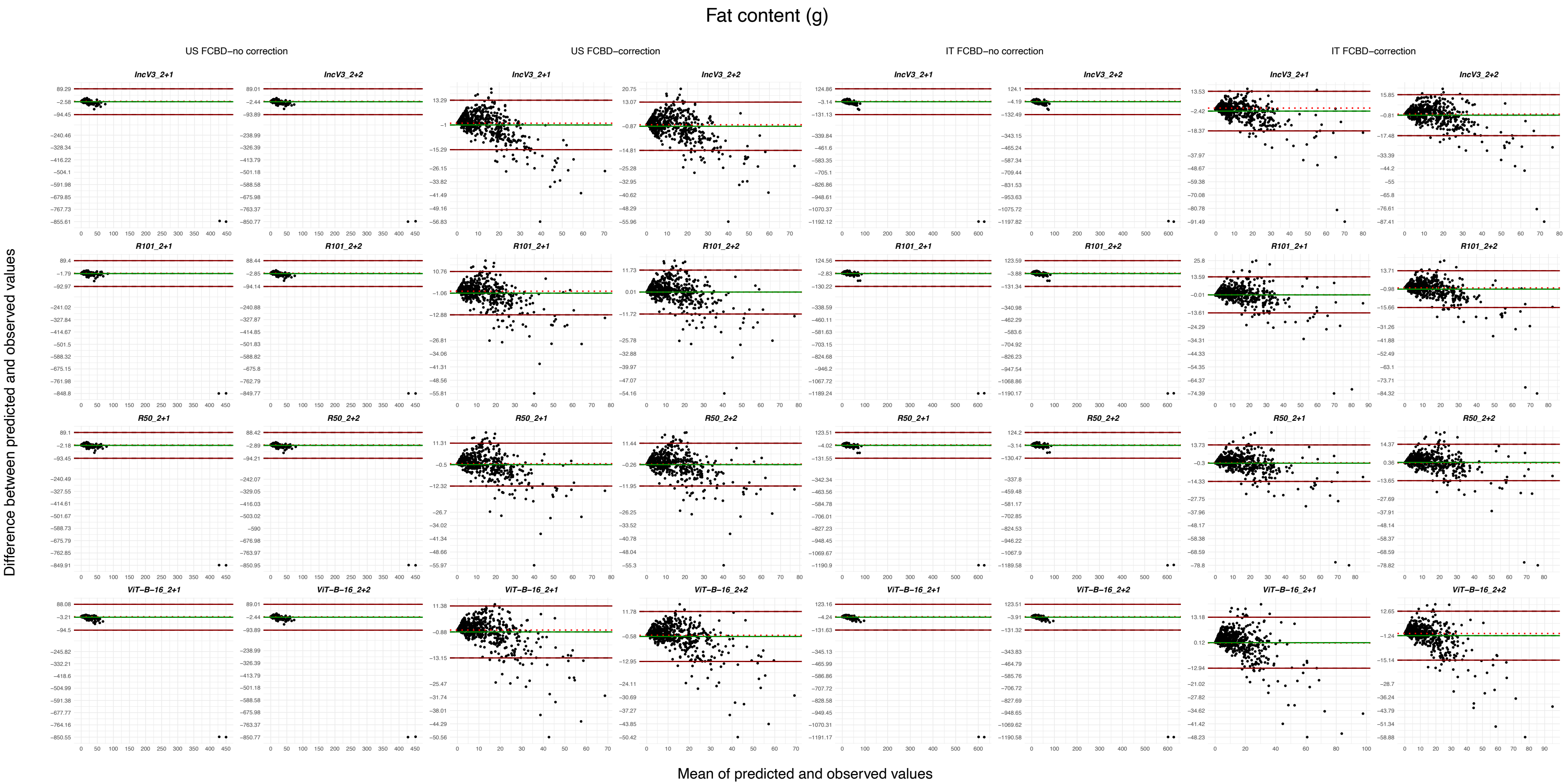
Fat content (g)
US FCBD–no correction
US FCBD–correction
IT FCBD–no correction
IT FCBD–correction
IncV3_2+1
IncV3_2+2
R101_2+1
R101_2+2
R50_2+1
R50_2+2
ViT–B–16_2+1
ViT–B–16_2+2
Difference between predicted and observed values
Mean of predicted and observed values

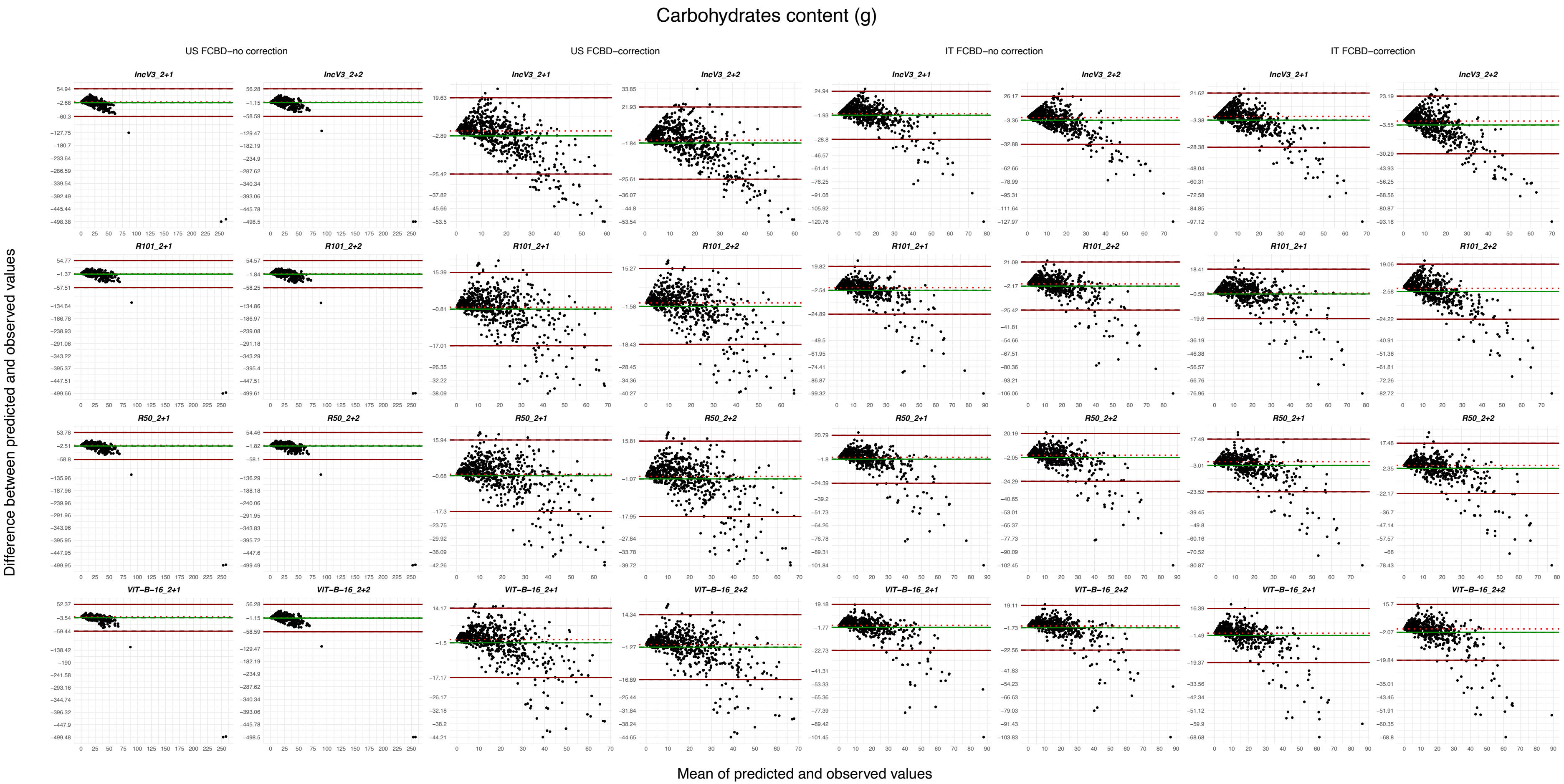
Carbohydrates content (g)
US FCBD–no correction
US FCBD–correction
IT FCBD–no correction
IT FCBD–correction
IncV3_2+1
IncV3_2+2
R101_2+1
R101_2+2
R50_2+1
R50_2+2
ViT–B–16_2+1
ViT–B–16_2+2
Difference between predicted and observed values
Mean of predicted and observed values
